# A comparative optimal control study of protecting susceptibles and isolating infecteds in SIR models

**DOI:** 10.1016/j.idm.2026.08.002

**Published:** 2026-09-07

**Authors:** Mohammed Elghandouri, Mostafa Adimy, Claudia Pio Ferreira

**Affiliations:** a Inria, CNRS, Centrale Lyon, INSA Lyon, Universite Claude Bernard Lyon 1, Université Jean Monnet, ICJ UMR5208, Villeurbanne, 69603, France; b São Paulo State University (UNESP), Institute of Biosciences, Botucatu, São Paulo, 18618-689, Brazil

**Keywords:** Ordinary differential equations, Optimal control, Disease control, Disease-free equilibrium, Endemic equilibrium, Basic reproduction number, Pontryagin's maximum Principle, 49J15, 34D23, 92D30

## Abstract

There are several approaches to controlling the spread of infectious diseases. Among the most widely used measures are the protection of susceptible individuals and the isolation of infected ones. This raises a critical question: which strategy, protecting susceptible individuals, isolating infected ones, or combining both, most effectively mitigates disease transmission? The work proposed here, address this question by analyzing protection and isolation strategies within the framework of the classical SIR (Susceptible-Infected-Recovered) epidemic model. Three scenarios are considered: (i) combined protection and isolation, (ii) protection alone, and (iii) isolation alone. Using Optimal Control Theory, we show that, in the short-term context of epidemic control, combining protection and isolation generally provides the most effective strategy under the considered conditions. However, when the combination is unavailable or unnecessary, isolation tends to outperform protection, particularly under challenging conditions such as high transmission rates or low recovery rates. Protection alone can still be effective, but primarily in settings where recovery rates are sufficiently high. These results contrast with those obtained from autonomous models, in which, in the long-term dynamics, protecting susceptible individuals proved to be more effective than isolating the infected.

## 1 Introduction

The control of epidemics and outbreaks remains an important and ongoing challenge. Since they have occurred for centuries and will continue to do so, it is essential to develop effective and innovative strategies to reduce or halt disease transmission. This issue is even more urgent today, as technological, economic, and social factors strongly influence public health responses and facilitate disease transmission through global mobility and trade. The goal is to protect populations while avoiding political, economic, or social crises by using efficient control measures.

In this scenario, mathematical approaches are essential for understanding how epidemics spread and how they can be controlled. Mathematical modeling and optimal control theory enable the simulation of epidemic dynamics, the evaluation of intervention strategies, and the design of cost-effective public health policies that account for real-world conditions. These methods were first applied to epidemics by pioneers such as D. Bernoulli (Bernoulli; Bernoulli, 1766), R. Ross (Ross, 1911), and W.O. Kermack and A.G. McKendrick (Ogilvy Kermack & McKendrick, 1927), laying the foundations for modern research (see also N.T.J. Bailey (Norman et al., 1957), W.H. Frost (Hampton Frost, 1976), K. Wickwire (Wickwire, 1977), D. Mollison (Mollison, 1977), R. Anderson (Anderson, 1991), B.T. Grenfell (Grenfell, 1992)).

The SIR (Susceptible-Infected-Recovered) models represent a fundamental class of epidemic models that form the basis for numerous studies seeking to understand and analyze the dynamics of epidemiological systems. These models are widely used to simulate the spread of infectious diseases within populations, enabling researchers and policymakers to assess the impact of interventions, forecast future trends, and design optimal control strategies. Foundational contributions include (Hethcote, 2000; Linda, 1994; Ogilvy Kermack & McKendrick, 1927), with modern extensions and generalized frameworks presented in (Brauer et al., 2012; Diekmann et al., 2010; Keeling & Rohani, 2008), and more recent adaptations addressing COVID-19 in (Abdoul Diop et al., 2025; Elghandouri et al., 2025; Li et al., 2020).

Common control strategies include quarantine, isolation, vaccination, closure of indoor settings, lockdowns, social distancing, mobility restrictions, testing, contact tracing, hospitalization, and other public health measures. Among these, the protection of susceptible individuals and the isolation of infected individuals are among the most frequently applied approaches. Protection measures may involve vaccination, closures, lockdowns, distancing, mobility restrictions, or any intervention that reduces the exposure of susceptibles to infection. Isolation strategies may include hospitalization, quarantine, or other targeted measures aimed at separating infected individuals from the general population, often combined with medical treatment to facilitate recovery.

The effects of protection and isolation in SIR models have been extensively studied in the literature; see, for example (Adimy et al., 2020; Balderrama et al., 2022; Bolzoni et al., 2021; Huang et al., 2021; Sturniolo et al., 2021; Wu et al., 2024), and references therein. For instance, in (Adimy et al., 2020), the authors study an SIR epidemic model incorporating an age-structured phase of protection with limited duration. Their model extends the classical Kermack-McKendrick framework (Ogilvy Kermack & McKendrick, 1927) by including a compartment for temporarily protected individuals. They analyze how this limited protection phase affects the dynamics of the SIR model, focusing in particular on the stability of the disease-free and disease-endemic equilibria.

In (Balderrama et al., 2022; Bolzoni et al., 2021), optimal control theory is applied through the implementation of a time-limited isolation strategy to minimize the number of infected individuals. These studies investigate how time-dependent isolation controls can help suppress disease transmission, with the Pontryagin Maximum Principle (PMP) and related techniques serving as key analytical tools. Similarly, interventions such as vaccination, testing, contact tracing, and isolation have been studied in (Sturniolo et al., 2021; Wu et al., 2024) to reduce the number of infections under endemic conditions. Vaccination is treated as a protection strategy, whereas testing and tracing act as pre-isolation measures. The effectiveness of each approach depends on the specific characteristics of the epidemic under study.

Despite these numerous contributions, an important question remains: which approach is most effective in curbing the spread of directly transmitted infectious diseases, protecting susceptible individuals, isolating infected individuals, or combining both strategies? This question was recently addressed in (Adimy et al., 2024), where the authors examined the relative effectiveness of protecting susceptible individuals versus isolating infected ones in an SIR-type epidemic model. They compared these two strategies using a hybrid delayed differential-difference SIR model derived from an age-structured system of partial differential equations, assuming constant rates for both protection and isolation. Their analysis showed that, in terms of long-term dynamics, protecting susceptible individuals was more effective than isolating the infected population alone.

This work provides the foundation for the present study. Building upon it, we apply optimal control theory to extend and refine the comparative analysis of protection and isolation strategies. Instead of relying on fixed intervention rates, as in (Adimy et al., 2024), we propose dynamic, time-dependent control strategies that adjust protection and isolation rates in response to the evolving epidemic. This approach is motivated by the practical nature of these interventions: protection measures such as indoor setting closures or lockdowns, and isolation measures such as quarantine, are rarely applied at uniform intensity over time. Implementing such measures at constant rates can be overly restrictive or unnecessarily costly, potentially causing significant economic and social disruption.

The basis of our study is the standard deterministic SIR epidemic model, and our comparative analysis is conducted progressively. We first formulate a controlled epidemic model by extending the classical SIR framework to include two additional compartments: *P*, representing the protected population, and *Q*, representing the isolated population. We then examine the case without control measures, focusing on the equilibrium points, the basic reproduction number $R_{0}$, and the long-term dynamics of the classical SIR model. This preliminary step is essential to understand the intrinsic dynamics of the model in the absence of interventions and serves as a baseline for evaluating the impact of control strategies. Next, we consider the case in which the protection and isolation rates are constant. In this setting, we analyze the equilibrium points, compute the controlled reproduction number $R_{c,0}$, and study the long-term dynamics of the system. This analysis lays the groundwork for the final and most important part of our study in which we address time-varying control functions. We formulate an optimal control problem to determine the time-dependent strategies for protection and isolation and compute the corresponding time-varying reproduction number $R_{c,0}\left(t\right)$. This approach enables us to evaluate the effectiveness and relevance of dynamic intervention strategies in the short-term context of epidemic control.

The paper is organized as follows. Section 2 introduces the formulation of the controlled epidemic model, while Section 3 analyzes the system without control measures. Section 4 addresses the implementation of protection and isolation strategies, covering (i) constant intervention rates, (ii) time-varying rates (controls), (iii) the characterization of optimal controls, and (iv) the time-dependent reproduction number. Numerical simulations are provided throughout to illustrate the analytical results. Concluding remarks are given in Section 5. The proofs of the main results are presented in detail in Appendix 6.

## 2 Formulation of the controlled model

Our optimal control approach introduces two time-dependent interventions to reduce disease transmission: the protection of susceptible individuals *S*(*t*) at rate *h*(*t*), with *h*_min_ ≤ *h*(*t*) ≤ *h*_max_, where *h*_min_ and *h*_max_ denote the minimum and maximum achievable levels of protection effort, reflecting limitations such as imperfect compliance and resource constraints, and the isolation of infected individuals *I*(*t*) at rate *k*(*t*), with *k*_min_ ≤ *k*(*t*) ≤ *k*_max_, where *k*_min_ and *k*_max_ represent the lowest and highest feasible isolation intensities, depending on factors such as detection efficiency, testing capacity, and healthcare infrastructure. The protection control *h*(*t*) may represent strict mobility restrictions, full lockdowns, mandatory vaccination, or other interventions that effectively shield susceptibles from infection. Protected individuals *P*(*t*) either stop their protection or retain only temporary immunity, and eventually return to the susceptible class through a reversion process occurring at rate *ℓ*(*t*), where *ℓ*_min_ ≤ *ℓ*(*t*) ≤ *ℓ*_max_, with *ℓ*_min_ and *ℓ*_max_ denoting the minimum and maximum rates of loss of protection or immunity, capturing both biological mechanisms (waning immunity) and socio-economic factors leading to the relaxation of protective measures. In particular, we assume *ℓ*_min_ > 0 to reflect the temporary nature of immunity or the economic and social constraints that prevent protection from being maintained indefinitely. An infected individual *I*(*t*) can be isolated once detected. Isolated individuals *Q*(*t*) experience an enhanced recovery rate $\bar{\alpha }\geq \alpha$ due to improved medical care, and a reduced transmission rate $\bar{\beta }\leq \beta$ resulting from limited contact, typically restricted to health-care workers under strict isolation conditions. All compartments are subject to a natural mortality rate *μ*.

The controlled epidemic dynamics extend the classical SIR model and are represented by the following system of ordinary differential equations:

(1) $$\left\{\begin{array}{ll} S^{\prime }\left(t\right) & =\mu N-\mu S\left(t\right)-\left(\beta I\left(t\right)+\bar{\beta }Q\left(t\right)\right)S\left(t\right)-h\left(t\right)S\left(t\right)+\ell \left(t\right)P\left(t\right),\\ I^{\prime }\left(t\right) & =-\mu I\left(t\right)-\alpha I\left(t\right)+\left(\beta I\left(t\right)+\bar{\beta }Q\left(t\right)\right)S\left(t\right)-k\left(t\right)I\left(t\right),\\ R^{\prime }\left(t\right) & =-\mu R\left(t\right)+\alpha I\left(t\right)+\bar{\alpha }Q\left(t\right),\\ P^{\prime }\left(t\right) & =-\mu P\left(t\right)-\ell \left(t\right)P\left(t\right)+h\left(t\right)S\left(t\right),\\ Q^{\prime }\left(t\right) & =-\mu Q\left(t\right)-\bar{\alpha }Q\left(t\right)+k\left(t\right)I\left(t\right),\\ N & =S\left(t\right)+I\left(t\right)+R\left(t\right)+P\left(t\right)+Q\left(t\right), \end{array}\right)$$

defined for *t* ∈ [0, *τ*] with *τ* > 0.

To simplify the analysis, we normalize the system by dividing all state variables by *N* and, for notational simplicity, we continue to denote them by *S*(*t*), *I*(*t*), *R*(*t*), *P*(*t*), and *Q*(*t*). Since the term $\left(\beta I\left(t\right)+\bar{\beta }Q\left(t\right)\right)S\left(t\right)$ is quadratic, we rescale the transmission coefficients as *β* ← *βN* and $\bar{\beta }\leftarrow \bar{\beta }N$ to preserve their epidemiological interpretation in the normalized system. The *R*(*t*) compartment can be omitted since the total population is constant. This reduces System (1) to the following set of ordinary differential equations:

(2) $$\left\{\begin{array}{ll} S^{\prime }\left(t\right) & =\mu -\mu S\left(t\right)-\left(\beta I\left(t\right)+\bar{\beta }Q\left(t\right)\right)S\left(t\right)-h\left(t\right)S\left(t\right)+\ell \left(t\right)P\left(t\right),\\ I^{\prime }\left(t\right) & =-\mu I\left(t\right)-\alpha I\left(t\right)+\left(\beta I\left(t\right)+\bar{\beta }Q\left(t\right)\right)S\left(t\right)-k\left(t\right)I\left(t\right),\\ P^{\prime }\left(t\right) & =-\mu P\left(t\right)-\ell \left(t\right)P\left(t\right)+h\left(t\right)S\left(t\right),\\ Q^{\prime }\left(t\right) & =-\mu Q\left(t\right)-\bar{\alpha }Q\left(t\right)+k\left(t\right)I\left(t\right). \end{array}\right)$$

Fig. 1 illustrates the proposed compartmental model, highlighting how protection and isolation modify the standard transmission pathways of the disease.

**Fig. 1:**
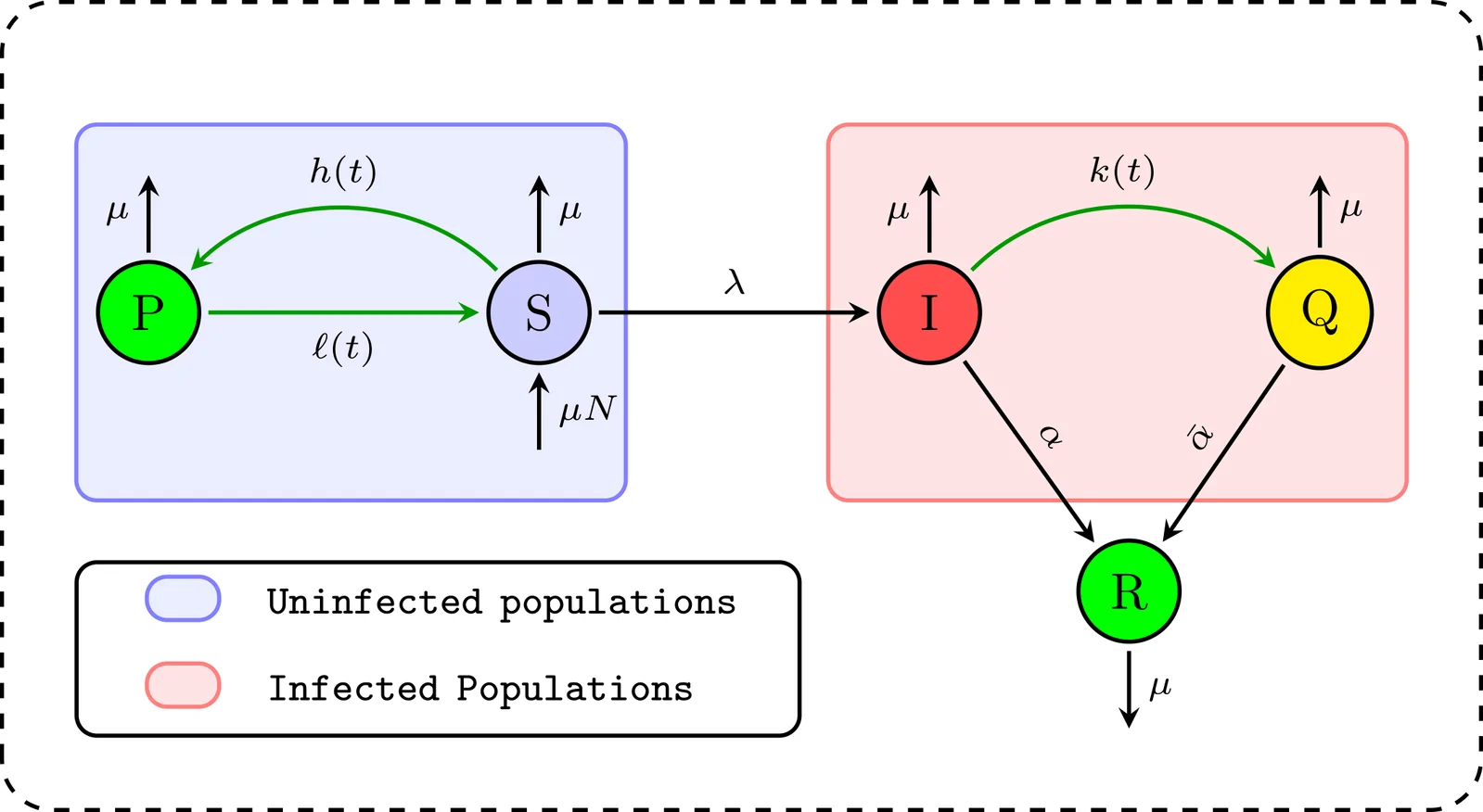
Scheme of the SIR epidemic model with protection *P* and isolation *Q* compartments. Susceptible individuals *S* are protected at rate *h*(*t*), while infected individuals *I* are isolated at rate *k*(*t*). The protected population returns to the susceptible class at rate *ℓ*(*t*). The other parameters *μ*, *β*, $\bar{\beta }$, *α*, and $\bar{\alpha }$, with $\bar{\beta }\leq \beta$ and $\alpha \leq \bar{\alpha }$, are time-independent and represent the mortality/natality rate, the transmission rates, and the transition rates to the recovered *R* compartment, respectively. The total population *N* is normalized to 1. The force of infection is given by $\lambda =\beta I+\bar{\beta }Q$, which represents the infection rates from infectious and isolated individuals, respectively.

We define the state space of System (2), which represents all admissible combinations of susceptible (*S*), infected (*I*), protected (*P*), and isolated (*Q*) populations, as the compact set:

(3) $$\Omega =\left\{{\left(S,I,P,Q\right)}^{T}\in R^{4}\;|\;0\leq S,I,P,Q\leq 1,\;\;S+I+P+Q\leq 1\right\}.$$

By applying the same idea as in Proposition 4.1 of (Haddad & Chellaboina, 2005), we show that Ω is positively invariant; that is, any trajectory starting in Ω remains in Ω for all *t* ≥ 0. This invariance ensures the well-posedness of the model, as it excludes inadmissible states such as negative populations or proportions exceeding unity.

Let us first analyze the uncontrolled model to identify the key transmission parameters.

## 3 In the absence of protection and isolation policies

This section provides the basis for the subsequent analysis by examining System (2) without protection or isolation controls. Specifically, we set *h*(*t*) = *k*(*t*) = 0 and *Q*(*t*) = *P*(*t*) = 0 for all *t* ≥ 0, reducing System (2) to the following classical simplified form:

(4) $$\left\{\begin{array}{ll} S^{\prime }\left(t\right) & =\mu -\mu S\left(t\right)-\beta I\left(t\right)S\left(t\right),\\ I^{\prime }\left(t\right) & =-\mu I\left(t\right)-\alpha I\left(t\right)+\beta I\left(t\right)S\left(t\right). \end{array}\right)$$

We focus on determining the equilibrium points, computing the basic reproduction number $R_{0}$, and analyzing the long-term dynamics of the system. These results are well established in the literature (see, *e.g.*, (Hethcote, 2000), [27, Section 3.3], [10, Chapter 9]), but we briefly recall and discuss the key findings, as understanding the system's dynamics is essential for the effective implementation of our control strategy.

To determine the equilibrium points (*S*∗, *I*∗) of System (4), we solve the following system:

$$\left\{\begin{array}{l} 0=\mu -\mu S^{*}-\beta S^{*}I^{*},\\ 0=-\left(\mu +\alpha \right)I^{*}+\beta S^{*}I^{*}, \end{array}\right)$$

This yields two equilibrium points:

• Disease-Free Equilibrium (DFE):

$${\bar{E}}_{0}^{*}≔\left(S^{*},I^{*}\right)=\left(1,0\right),$$

which always exists and represents a population entirely free of infection.

• Endemic Equilibrium (EE):

$${\bar{E}}_{d}^{*}≔\left(S^{*},I^{*}\right)=\left(\frac{\alpha +\mu }{\beta },\;\frac{\mu }{\alpha +\mu }-\frac{\mu }{\beta }\right),$$

which exists only if

$$\beta >\alpha +\mu \;\text{which is equivalent to}\;R_{0}≔\frac{\beta }{\alpha +\mu }>1.$$

In epidemiological terms, this condition means that the transmission rate must be sufficiently large relative to the recovery and natural mortality rates for the disease to persist in the population.

We recall the classical results:

• If $R_{0}<1$, the population remains free of disease, and the system trajectories converge asymptotically to the disease-free equilibrium $E_{0}^{*}$ from any admissible initial condition.
• If $R_{0}>1$, the trajectories converge asymptotically to the disease-endemic equilibrium $E_{d}^{*}$, indicating that the disease persists and must be controlled; see, for instance, (Hethcote, 2000; Thieme, 2018; Van den Driessche & Watmough, 2002).

Fig. 2 illustrates the phase-plane trajectories of the infected fraction *I* as a function of the susceptible fraction *S* over time for three transmission rates: *β* = 4.95 × 10^−4^, *β* = 1.95 × 10^−3^, and *β* = 3.81 × 10^−3^. For *β* = 4.95 × 10^−4^, the basic reproduction number is $R_{0}\approx 0.90<1$, implying that only the disease-free equilibrium $E_{0}^{*}$ exists and is globally asymptotically stable. In contrast, for *β* = 1.95 × 10^−3^ and *β* = 3.81 × 10^−3^, we have $R_{0}\approx 3.55>1$ and $R_{0}\approx 6.93>1$, respectively, confirming the global stability of the disease-endemic equilibrium $E_{d}^{*}$ in both cases. The trajectories in Fig. 2 clearly illustrate these behaviors: in all scenarios, the system converges to the corresponding equilibrium for any positive initial condition (*S*_0_, *I*_0_). Moreover, higher transmission rates result in equilibria with fewer susceptibles and more infected individuals, emphasizing the critical role of *β* in shaping epidemic outcomes, given that the other parameters that appear at *R*_0_ expression are fixed.

**Fig. 2:**
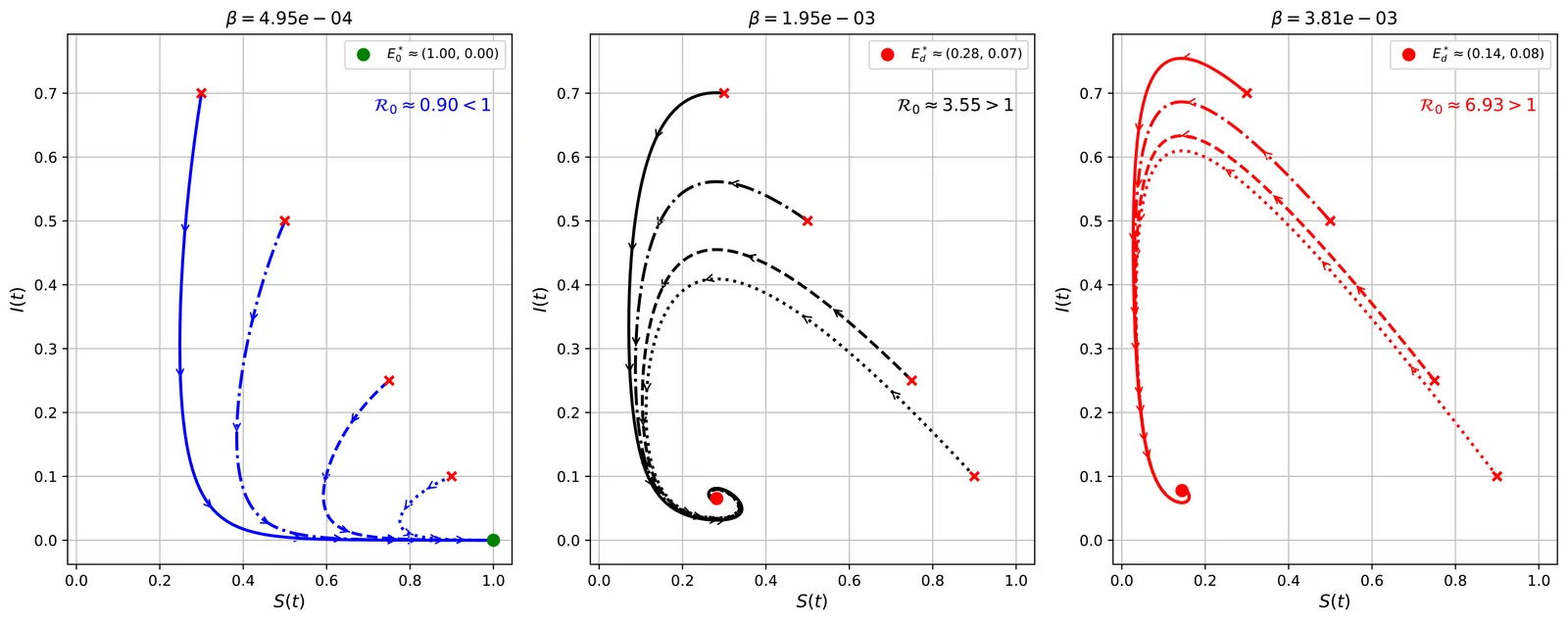
Phase-plane trajectories of the infected fraction *I* versus the susceptible fraction *S* for three transmission rates: *β* = 4.95 × 10^−4^, *β* = 1.95 × 10^−3^, and *β* = 3.81 × 10^−3^. Equilibrium points $E_{0}^{*}$ and $E_{d}^{*}$ are shown for each case. The other parameter values are *μ* = 5 × 10^−5^ and *α* = 5 × 10^−4^. Four different initial conditions are used in each panel.

Fig. 3 illustrates the phase-plane trajectories of the infected fraction *I* as a function of the susceptible fraction *S* over time for three values of the recovery rate: *α* = 5 × 10^−4^, *α* = 2 × 10^−3^, and *α* = 3.9 × 10^−3^. For *α* = 5 × 10^−4^ and *α* = 2 × 10^−3^, we obtain $R_{0}\approx 6.93>1$ and $R_{0}\approx 1.86>1$, respectively, indicating the existence and global asymptotic stability of the disease-endemic equilibrium $E_{d}^{*}$ for both values of *α*. In contrast, for *α* = 3.90 × 10^−3^, we have $R_{0}\approx 0.96<1$, which implies that only the disease-free equilibrium $E_{0}^{*}$ exists and is globally asymptotically stable. In all scenarios, the trajectories converge to their corresponding equilibrium points for any positive initial condition (*S*_0_, *I*_0_). Smaller values of *α* yield equilibrium points with lower levels of susceptibles and higher levels of infecteds, highlighting the critical role of the recovery rate in shaping the epidemic outcome, given that the other parameters appearing in the expression of $R_{0}$ are fixed.

**Fig. 3:**
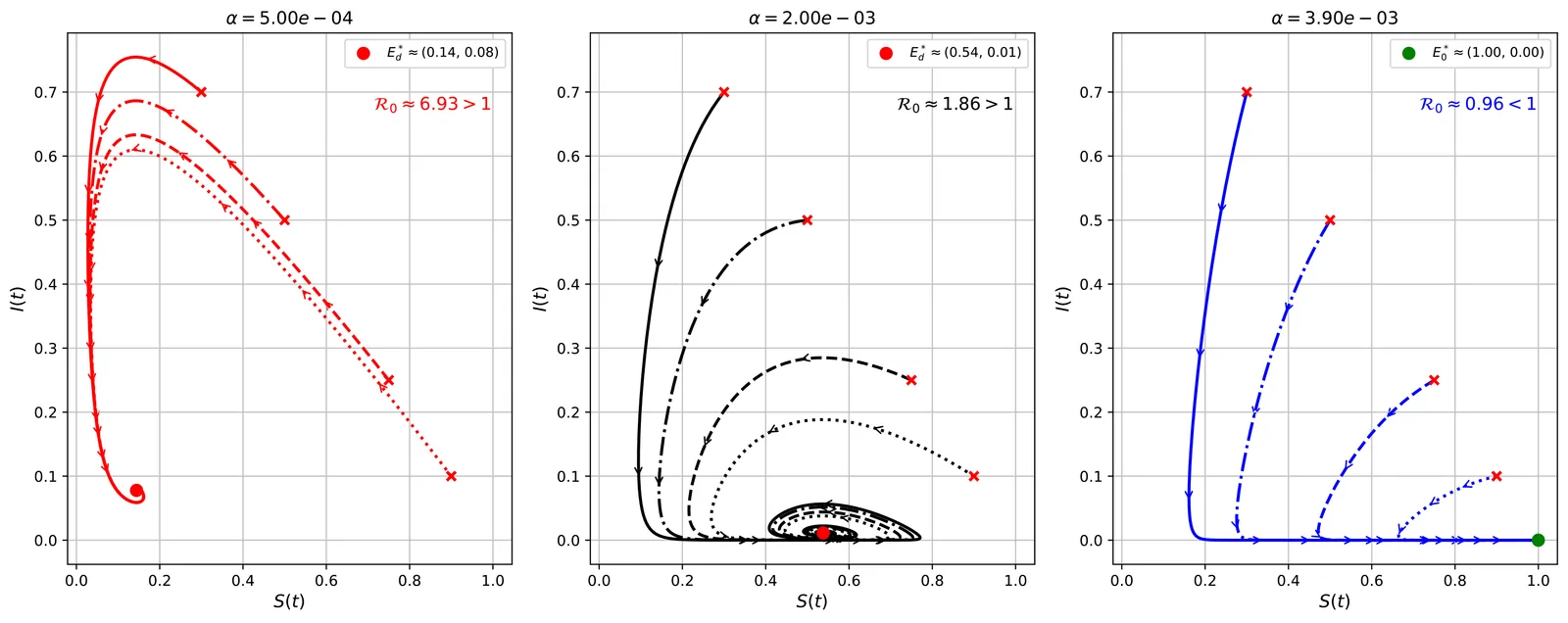
Phase-plane trajectories of the infected fraction *I* versus the susceptible fraction *S* for three recovery rates: *α* = 5 × 10^−4^, *α* = 2 × 10^−3^, and *α* = 3.90 × 10^−3^. Equilibrium points $E_{0}^{*}$ and $E_{d}^{*}$ are indicated for each case. The other parameters are *μ* = 5 × 10^−5^ and *β* = 3.81 × 10^−3^. Four different initial conditions are used in each pannel.

Hence, reducing the transmission rate *β* or increasing the recovery rate *α* can impact disease transmission dynamics, ultimately leading to disease eradication. Such interventions are therefore critical for controlling the transmission of the disease. This has been investigated in several studies (see (Fleming & Rishel, 2012; Lenhart & Workman, 2007; Sharomi & Malik, 2017; Zaman et al., 2009)), where control theory is applied by modeling *β* and *α* as control-dependent functions, *β*(*u*) and *α*(*v*), with *u* and *v* representing the implemented strategies.

Indirect control strategies can also be effective. In a non-autonomous model, this can be achieved by reducing the fractions of susceptible and infected individuals. Even with a constant transmission rate *β*, the product *βS*(*t*)*I*(*t*) can decrease over time, thereby limiting disease transmission. In the following sections, we apply optimal control theory to examine how such measures can be effectively implemented. Specifically, we model protection and isolation policies as control functions acting on the susceptible and infected populations, respectively. These interventions are designed to reduce the effective transmission rate and can promote faster recovery by pharmacological procedures, such as administering drugs, acting on infected individuals. Through a comparative analysis, we determine which approach, protection, isolation, or a combination of both, is most effective in limiting disease spread.

## 4 In the presence of protection and isolation policies

We assume $R_{0}>1$, indicating that the infection persists and must be controlled. We then turn our attention to System (2), where protection of susceptibles and isolation of infecteds are introduced as control measures. The following subsections investigate the effects of both static and time-dependent control strategies on the system's dynamics.

### 4.1 Constant rates of protection and isolation

In this subsection, we analyze System (2) under the assumption of constant protection and isolation rates. Specifically, we set $h\left(t\right)≔\bar{h}$, $\ell \left(t\right)≔\bar{\ell }$, and $k\left(t\right)≔\bar{k}$ for all *t* ∈ [0, *τ*], where $h_{\min}\leq \bar{h}\leq h_{\max}$, $\ell_{\min}\leq \bar{\ell }\leq \ell_{\max}$, and $k_{\min}\leq \bar{k}\leq k_{\max}$. Under these assumptions, System (2) reduces to the following form, for *t* ∈ [0, *τ*]:

(5) $$\left\{\begin{array}{ll} S^{\prime }\left(t\right) & =\mu -\mu S\left(t\right)-\left(\beta I\left(t\right)+\bar{\beta }Q\left(t\right)\right)S\left(t\right)-\bar{h}S\left(t\right)+\bar{\ell }P\left(t\right),\\ I^{\prime }\left(t\right) & =-\mu I\left(t\right)-\alpha I\left(t\right)+\left(\beta I\left(t\right)+\bar{\beta }Q\left(t\right)\right)S\left(t\right)-\bar{k}I\left(t\right),\\ P^{\prime }\left(t\right) & =-\mu P\left(t\right)+-\bar{\ell }P\left(t\right)+\bar{h}S\left(t\right),\\ Q^{\prime }\left(t\right) & =-\mu Q\left(t\right)+\bar{\alpha }Q\left(t\right)+\bar{k}I\left(t\right). \end{array}\right)$$

By direct calculation, we show that System (5) has two equilibrium points:

• Disease-Free Equilibrium (DFE), given by

$$E_{c,0}^{*}=\left(\frac{\mu +\bar{\ell }}{\mu +\bar{\ell }+\bar{h}},0,\frac{\bar{h}}{\mu +\bar{\ell }+\bar{h}},0\right).$$

• Endemic Equilibrium (EE), given by

$$E_{c,d}^{*}=\left(S^{*},I^{*},P^{*},Q^{*}\right),$$

where

$$S^{*}=\frac{\left(\mu +\bar{\alpha }\right)\left(\mu +\alpha +\bar{k}\right)}{\beta \left(\mu +\bar{\alpha }\right)+\bar{\beta }\bar{k}},\;I^{*}=\frac{\mu }{\mu +\alpha +\bar{k}}-\frac{\mu \left(\mu +\bar{\alpha }\right)\left(\mu +\bar{\ell }+\bar{h}\right)}{\left(\mu +\bar{\ell }\right)\left(\beta \left(\mu +\bar{\alpha }\right)+\bar{\beta }\bar{k}\right)},$$

$$P^{*}=\frac{\bar{h}}{\mu +\bar{\ell }}S^{*},\;\text{and}\;Q^{*}=\frac{\bar{k}}{\mu +\bar{\alpha }}I^{*}.$$

Using the next-generation matrix method, we obtain the basic reproduction number of System (5), denoted by $R_{c,0}$, as

$$R_{c,0}=\frac{\beta \left(\mu +\bar{\ell }\right)}{\left(\mu +\bar{\ell }+\bar{h}\right)\left(\mu +\alpha +\bar{k}\right)}+\frac{\bar{\beta }\left(\mu +\bar{\ell }\right)\bar{k}}{\left(\mu +\bar{\ell }+\bar{h}\right)\left(\mu +\bar{\alpha }\right)\left(\mu +\alpha +\bar{k}\right)}.$$

If $R_{c,0}<1$, only the disease-free equilibrium (DFE) exists and is asymptotically stable, implying that the population remains free from infection. However, if $R_{c,0}>1$, the endemic equilibrium (EE) exists, and the dynamics of System (5) converge to it, indicating that the infection becomes endemic and that control measures must be actively enforced to manage the outbreak.

$R_{c,0}$ can also be expressed in terms of $R_{0}$ (the basic reproduction number of System (4)) as

$$R_{c,0}=f\left(\bar{h},\bar{\ell },\bar{k}\right)\times R_{0},$$

where

$$f\left(\bar{h},\bar{\ell },\bar{k}\right)=\frac{\mu +\bar{\ell }}{\mu +\bar{\ell }+\bar{h}}\times \frac{\mu +\alpha }{\mu +\alpha +\bar{k}}\times \left(1+\frac{\bar{\beta }\bar{k}}{\beta \left(\mu +\bar{\alpha }\right)}\right).$$

Since isolation both increases recovery $\left(\bar{\alpha }\geq \alpha \right)$ and reduces infection $\left(\bar{\beta }\leq \beta \right)$, we obtain

$$\frac{\partial f\left(\bar{h},\bar{\ell },\bar{k}\right)}{\partial \bar{k}}=\frac{\mu +\bar{\ell }}{\mu +\bar{\ell }+\bar{h}}\times \frac{\mu +\alpha }{{\left(\mu +\alpha +\bar{k}\right)}^{2}}\times \left(-1+\frac{\bar{\beta }}{\beta }\times \frac{\mu +\alpha }{\mu +\bar{\alpha }}\right)\leq 0,$$

$$\frac{\partial f\left(\bar{h},\bar{\ell },\bar{k}\right)}{\partial \bar{h}}=-\frac{\mu +\bar{\ell }}{{\left(\mu +\bar{\ell }+\bar{h}\right)}^{2}}\times \frac{\mu +\alpha }{\mu +\alpha +\bar{k}}\times \left(1+\frac{\bar{\beta }\bar{k}}{\beta \left(\mu +\bar{\alpha }\right)}\right)\leq 0,$$

and

$$\frac{\partial f\left(\bar{h},\bar{\ell },\bar{k}\right)}{\partial \bar{\ell }}=\frac{\bar{h}}{{\left(\mu +\bar{\ell }+\bar{h}\right)}^{2}}\times \frac{\mu +\alpha }{\mu +\alpha +\bar{k}}\times \left(1+\frac{\bar{\beta }\bar{k}}{\beta \left(\mu +\bar{\alpha }\right)}\right)\geq 0.$$

It follows that $f\left(\bar{h},\bar{\ell },\bar{k}\right)$ is decreasing in $\bar{h}$ and $\bar{k}$, and increasing in $\bar{\ell }$. In particular, we have

$$0\leq f\left(\bar{h},\bar{\ell },\bar{k}\right)\leq f\left(0,\bar{\ell },0\right)=1.$$

Increasing the protection rate $\bar{h}$ and isolation rate $\bar{k}$, while decreasing the return rate $\bar{\ell }$, reduces the controlled reproduction number $R_{c,0}$ and thereby facilitates the control or possible elimination of the disease.

Consider the case where $\bar{k}=0$, which represents the absence of isolation measures for infected individuals. The controlled reproduction number then takes the form:

$$R_{c,0}=f\left(\bar{h},\bar{\ell },0\right)\times R_{0},$$

where

$$f\left(\bar{h},\bar{\ell },0\right)=\frac{\mu +\bar{\ell }}{\mu +\bar{\ell }+\bar{h}}.$$

For effective control (*i.e.*, $R_{c,0}<1$), the following inequality must be satisfied, noting that we are considering the case $R_{0}>1$:

(6) $$\left(\mu +\bar{\ell }\right)\left(R_{0}-1\right)<\bar{h}\leq h_{\max}.$$

In order to determine a value of $\bar{h}$ satisfying (6), the following condition must hold:

(7) $$R_{0}<1+\frac{h_{\max}}{\mu +\bar{\ell }}.$$

Similarly, for the return control $\bar{\ell }$, the following condition must be satisfied:

(8) $$R_{0}<1+\frac{\bar{h}+\ell_{\min}}{\mu }.$$

Assume that

$$R_{0}<\min\left(1+\frac{h_{\max}}{\mu +{\bar{\ell }}_{\max}},\;1+\frac{h_{\min}+\ell_{\min}}{\mu }\right).$$

Then both conditions (7) and (8) are satisfied. The upper bound

$$B_{\text{up}}≔\min\left(1+\frac{h_{\max}}{\mu +\ell_{\max}},\;1+\frac{h_{\min}+\ell_{\min}}{\mu }\right)$$

is critical: If $R_{0}>B_{\text{up}}$, protection measures applied to susceptibles alone are insufficient to control the outbreak.

For the particular case $\bar{\ell }=h_{\max}-\bar{h}$, we obtain

$$R_{c,0}=\left(1-\frac{\bar{h}}{\mu +h_{\max}}\right)R_{0}.$$

Thus, $R_{c,0}<1$ is equivalent to

(9) $$\frac{\left(\mu +h_{\max}\right)\left(R_{0}-1\right)}{R_{0}}<\bar{h}\leq h_{\max},$$

which requires the condition

(10) $$R_{0}<1+\frac{h_{\max}}{\mu }.$$

Then, for $\bar{k}=0$ and $\bar{\ell }=h_{\max}-\bar{h}$, the upper bound becomes:

$$B_{\text{up}}≔1+\frac{h_{\max}}{\mu }.$$

Consider now the case $\bar{h}=0$ (and, intuitively, $\bar{\ell }=0$ as well). This represents a situation with no protection for susceptibles. In this case, the controlled reproduction number becomes

$$R_{c,0}=f\left(0,0,\bar{k}\right)\times R_{0},$$

with

$$f\left(0,0,\bar{k}\right)=\frac{\mu +\alpha }{\mu +\alpha +\bar{k}}\left(1+\frac{\bar{\beta }\bar{k}}{\beta \left(\mu +\bar{\alpha }\right)}\right).$$

To achieve effective control in this scenario, the following condition must be satisfied:

(11) $$\frac{\beta \left(\mu +\bar{\alpha }\right)\left(\mu +\alpha \right)\left(R_{0}-1\right)}{\beta \left(\mu +\bar{\alpha }\right)-\bar{\beta }\left(\mu +\alpha \right)}<\bar{k}\leq k_{\max}.$$

This follows from the inequality $R_{c,0}<1$ with $\bar{k}\leq k_{\max}$. For condition (11) to be satisfied for some $\bar{k}\leq k_{\max}$, it is necessary that the following condition holds:

(12) $$R_{0}<1+\frac{k_{\max}}{\beta \left(\mu +\bar{\alpha }\right)\left(\mu +\alpha \right)}.$$

Then, the upper bound becomes

$$B_{\text{up}}≔1+\frac{k_{\max}}{\beta \left(\mu +\bar{\alpha }\right)\left(\mu +\alpha \right)}.$$

If $R_{0}$ exceeds this upper bound, isolation efforts targeting infecteds alone are no longer sufficient to control the outbreak.

Fig. 4 illustrates the behavior of the controlled reproduction number $R_{c,0}$ as a function of $\left(\bar{h},\bar{k}\right)$ in the particular case where $\bar{\ell }=h_{\max}-\bar{h}=1-\bar{h}$ (assuming *h*_max_ = 1). Under the chosen parameter set, the basic reproduction number is $R_{0}\approx 6.927>1$. As shown, there exists a threshold condition that must be satisfied for the protection of susceptibles and isolation of infected individuals to effectively control the spread of the disease, *i.e.*, to ensure that $R_{c,0}<1$. This threshold corresponds to the projection onto the $\bar{h}$- and $\bar{k}$-axes of the contour line defined by $R_{c,0}\left(\bar{h},\bar{k}\right)=1$.

**Fig. 4:**
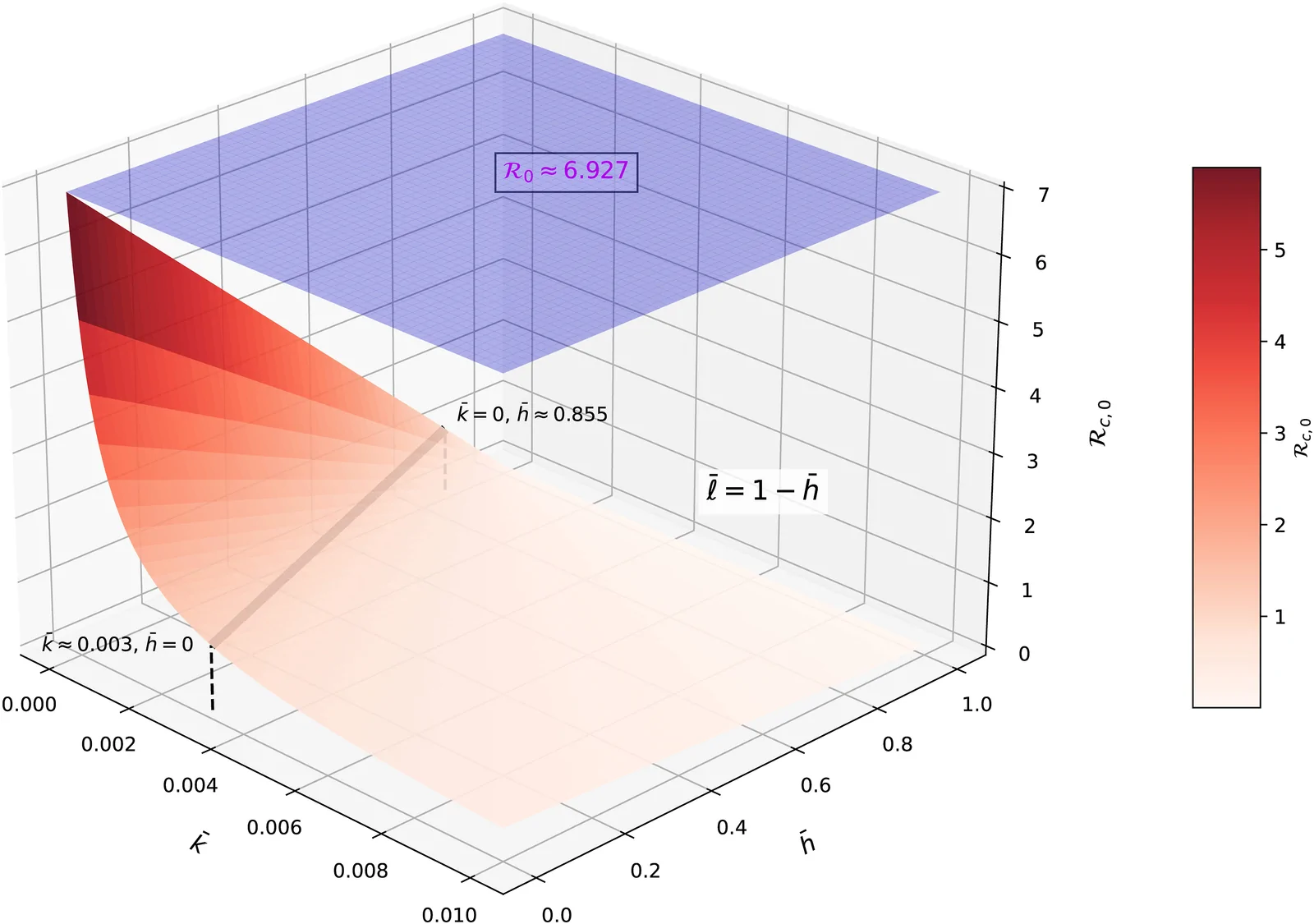
Dynamics of the controlled reproduction number $R_{c,0}$ as a function of $\bar{h}$ and $\bar{k}$ in the particular case where $\bar{\ell }=h_{\max}-\bar{h}=1-\bar{h}$, with *h*_max_ = 1. The remaining parameters are fixed as follows: *h*_min_ = 0, *k*_min_ = 0, *k*_max_ = 0.01, *μ* = 5 × 10^−5^, *β* = 3.81 × 10^−3^, $\bar{\beta }=2.8\times 10^{-8}$, *α* = 5 × 10^−4^, and $\bar{\alpha }=1.9\times 10^{-3}$. Under these parameter values, the basic reproduction number is $R_{0}=6.927>1$.

In particular, when there is no isolation $\left(\bar{k}=0\right)$, the required protection threshold is

$${\bar{h}}_{*}=\frac{\left(\mu +h_{\max}\right)\left(R_{0}-1\right)}{R_{0}}\approx 0.855,$$

which is the left bound of $\bar{h}$ in (9). When there is no protection $\left(\bar{h}=0\right)$, the required isolation threshold is

$${\bar{k}}_{*}=\frac{\beta \left(\mu +\bar{\alpha }\right)\left(\mu +\alpha \right)\left(R_{0}-1\right)}{\beta \left(\mu +\bar{\alpha }\right)-\bar{\beta }\left(\mu +\alpha \right)}\approx 0.003,$$

which is the left bound of $\bar{k}$ in (11). In these two cases, the conditions (10) and (12) are satisfied. This implies that it remains feasible to control the spread of the disease either by implementing isolation of infected individuals without protection of susceptibles $\left(\bar{h}=0\right)$, or by applying protection of susceptibles without isolation control $\left(\bar{k}=0\right)$.

Fig. 5 shows the dynamics of the susceptible (*S*), infected (*I*), and protected (*P*) populations, together with the phase-plane trajectory *I*(*S*), for $\bar{k}=0$ (no isolation) and two distinct protection scenarios: $\bar{h}=0.5<{\bar{h}}_{*}$ and $\bar{h}=0.86>{\bar{h}}_{*}$, with $\bar{\ell }=1-\bar{h}$. The chosen model parameters yield $R_{0}=\frac{\beta }{\mu +\alpha }\approx 6.927$; therefore, in the absence of control measures, the disease will persist.

**Fig. 5:**
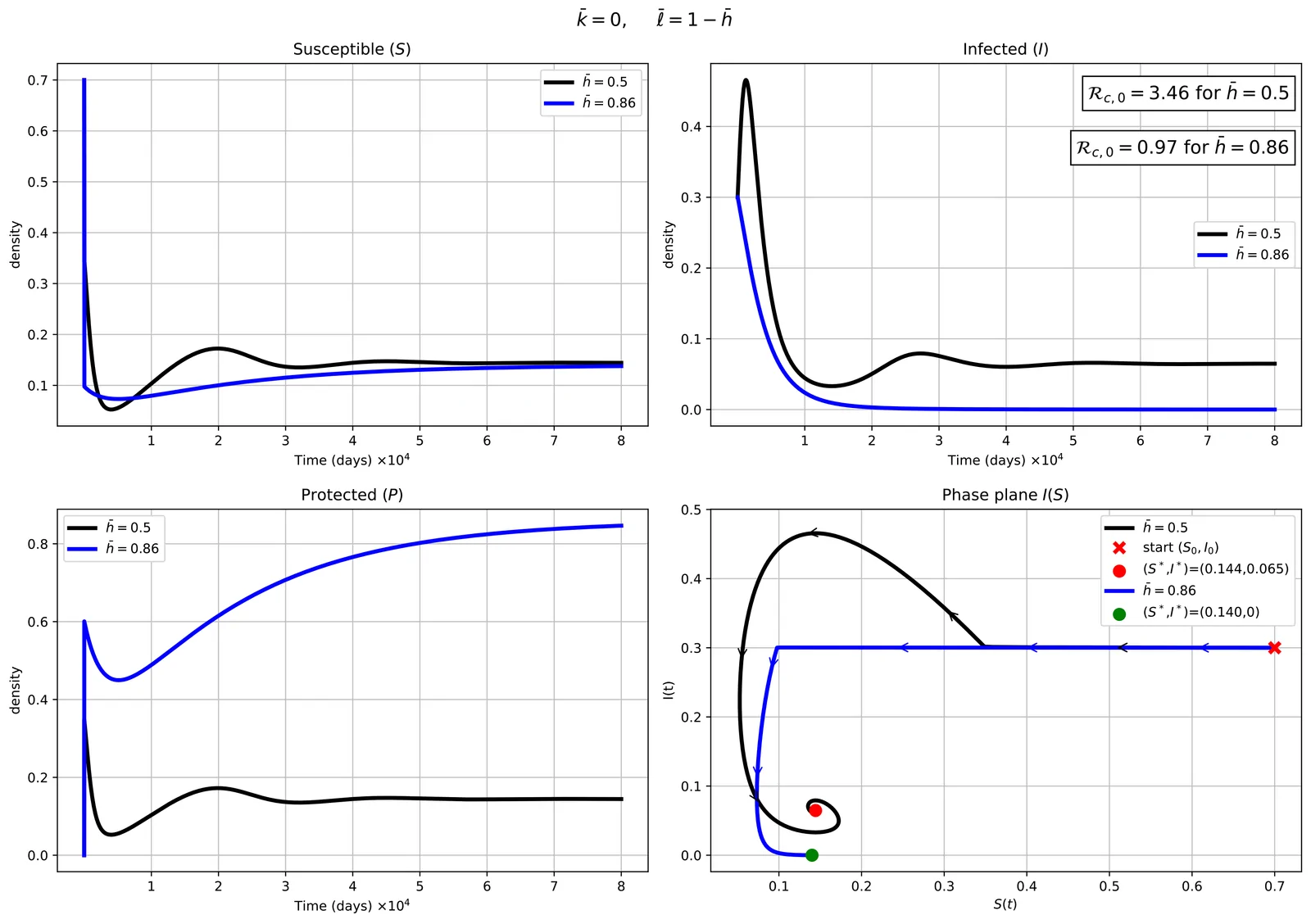
Dynamics of the susceptible (*S*), infected (*I*), and protected (*P*) populations over time for $\bar{k}=0$ and two protection scenarios ($\bar{h}=0.5<{\bar{h}}_{*}$ and $\bar{h}=0.86>{\bar{h}}_{*}$), where $\bar{\ell }=h_{\max}-\bar{h}=1-\bar{h}$ as *h*_max_ = 1. The rest of the parameters are fixed at: *h*_min_ = 0, *k*_min_ = 0, *k*_max_ = 0.01, *μ* = 5 × 10^−5^, *β* = 3.81 × 10^−3^, $\bar{\beta }=2.8\times 10^{-8}$, *α* = 5 × 10^−4^, and $\bar{\alpha }=1.9\times 10^{-3}$. Initial densities are: *S*_0_ = 0.7, *I*_0_ = 0.3, *P*_0_ = 0, and *Q*_0_ = 0. The corresponding phase-plane trajectories of *I* versus *S* is also shown.

For $\bar{h}=0.5$ (represented by the black lines in the subfigures of Fig. 5), the system dynamics converge toward the disease-endemic equilibrium point $E_{d}^{*}$ (marked by the red point in the phase-plane subfigure of Fig. 5).This shows that protection at the rate $\bar{h}=0.5$ is insufficient to effectively halt disease transmission. Furthermore, condition (6) is not satisfied for $\bar{h}=0.5$, and the controlled reproduction number is $R_{c,0}=f\left(0.5,0.5,0\right)\times R_{0}\approx 3.46>1$. This confirms that the disease would persist, highlighting the need for enhanced epidemic control strategies to effectively curb its transmission.

For $\bar{h}=0.86$ (represented by the blue lines in the subfigures of Fig. 5), the system dynamics converge toward the disease-free equilibrium point $E_{0}^{*}$, marked by the green point in the phase-plane subfigure. Here, condition (6) is satisfied, and the controlled reproduction number is reduced to $R_{c,0}=f\left(0.86,0.14,0\right)\times R_{0}\approx 0.97<1$. This confirms that protection at this rate successfully mitigates the spread of the disease.

Fig. 6 illustrates the dynamics of the susceptible (*S*), infected (*I*), and isolated (*Q*) populations, along with the phase-plane plot *I*(*S*), for $\bar{h}=0$ (no protection implemented) and two isolation scenarios: $\bar{k}=0.001<{\bar{k}}_{*}$ and $\bar{k}=0.004>{\bar{k}}_{*}$, with $\bar{\ell }=h_{\max}-\bar{h}=1$. As in the scenario before, the basic reproduction number is $R_{0}\approx 6.927>1$, indicating that the disease persists if no control measures are applied. Note that for $\bar{h}=0$, the system dynamics are independent of $\bar{\ell }$, making the condition $\bar{\ell }=h_{\max}-\bar{h}=1$ irrelevant.

**Fig. 6:**
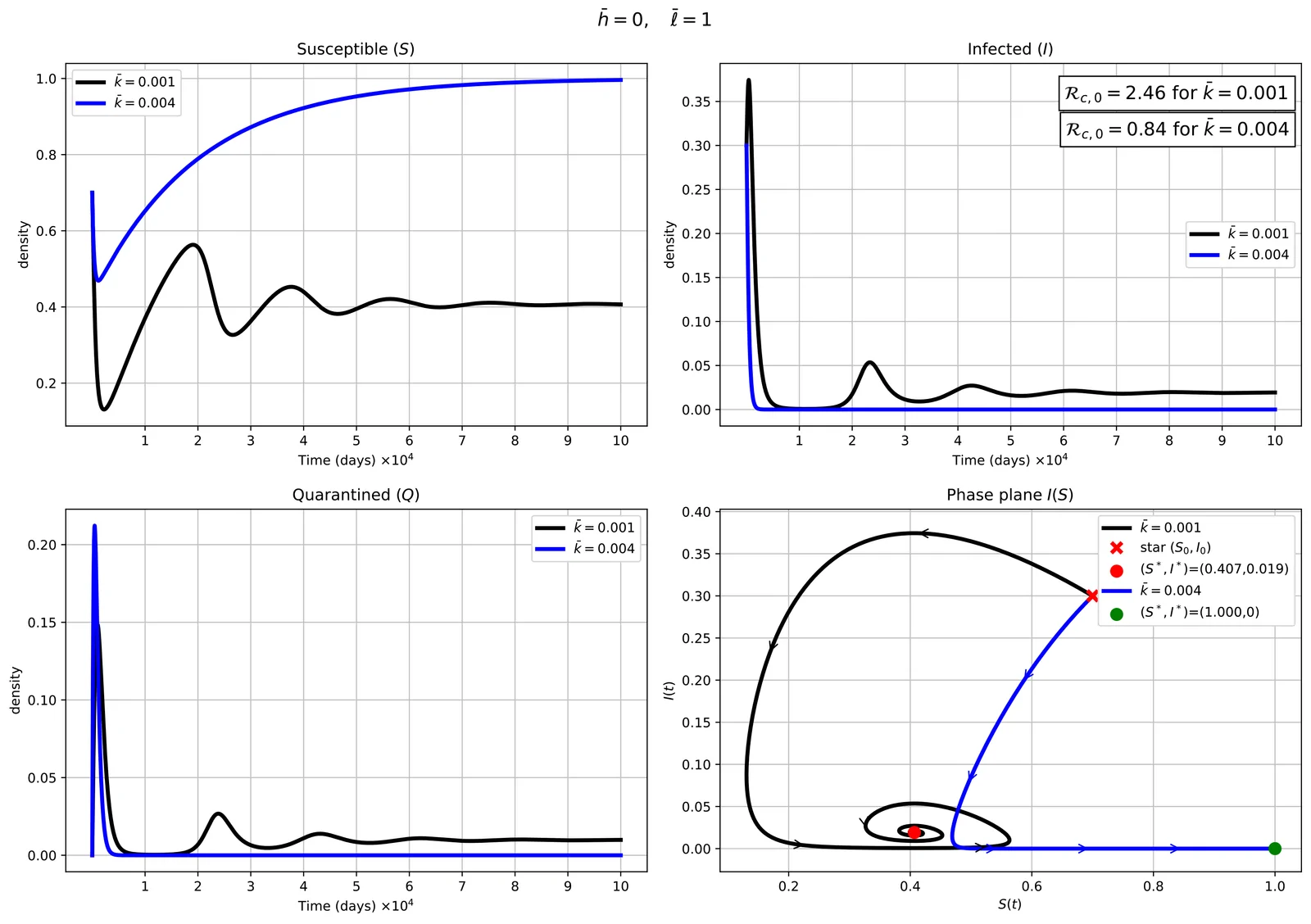
Dynamics of the susceptible (*S*), infected (*I*), and protected (*P*) populations over time for $\bar{h}=0$ and two isolation scenarios ($\bar{k}=0.003<{\bar{k}}_{*}$ and $\bar{k}=0.004>{\bar{k}}_{*}$), where $\bar{\ell }=h_{\max}-\bar{h}=1-\bar{h}$ as *h*_max_ = 1. The rest of the parameters are fixed at: *h*_min_ = 0, *k*_min_ = 0, *k*_max_ = 0.01, *μ* = 5 × 10^−5^, *β* = 3.81 × 10^−3^, $\bar{\beta }=2.8\times 10^{-8}$, *α* = 5 × 10^−4^, and $\bar{\alpha }=1.9\times 10^{-3}$. Initial densities are: *S*_0_ = 0.7, *I*_0_ = 0.3, *P*_0_ = 0, and *Q*_0_ = 0. The corresponding phase-plane trajectories of *I* versus *S* is also shown.

For $\bar{k}=0.001$ (represented by the black curves in Fig. 6), the system dynamics converge to the disease-endemic equilibrium point $E_{d}^{*}$ (marked by the red point in the phase-plane subfigure). Here, the controlled reproduction number is reduced to $R_{c,0}=f\left(0,1,0.001\right)\times R_{0}\approx 2.46>1$. This indicates that the isolation rate $\bar{k}=0.001$ is insufficient to control the epidemic, as it lies below the critical threshold ${\bar{k}}_{*}=0.003$. To effectively mitigate the outbreak, the isolation measures must be reinforced such that $\bar{k}>{\bar{k}}_{*}$.

For $\bar{k}=0.004$ (represented by the blue curves in Fig. 6), the system dynamics converge to the disease-free equilibrium point $E_{0}^{*}$, marked by the green point in the phase-plane subfigure. Here, the controlled reproduction number is reduced to $R_{c,0}=f\left(0,1,0.004\right)\times R_{0}\approx 0.84<1$. This demonstrates that an isolation rate of $\bar{k}=0.004$ is sufficient to suppress the epidemic, as it exceeds the critical threshold ${\bar{k}}_{*}\approx 0.003$. The results confirm that when no protection measures are implemented $\left(\bar{h}=0\right)$, maintaining $\bar{k}>{\bar{k}}_{*}$ is necessary to ensure disease eradication.

The numerical simulations and threshold analysis highlight the critical role of protection $\left(\bar{h}\right)$ and isolation $\left(\bar{k}\right)$ measures in controlling epidemic spread. Our results reveal two distinct scenarios:

(i) Isolation-free scenario $\left(\bar{k}=0\right)$: Disease eradication requires protection rates exceeding the critical threshold ${\bar{h}}_{*}\approx 0.855$. Below this threshold (*e.g.*, $\bar{h}=0.5$), the system converges to an endemic equilibrium $E_{d}^{*}$, while above it (*e.g.*, $\bar{h}=0.86$), the disease-free equilibrium $E_{0}^{*}$ is achieved.
(ii) Protection-free scenario $\left(\bar{h}=0\right)$: Effective outbreak suppression necessitates isolation rates surpassing ${\bar{k}}_{*}\approx 0.003$. Simulations confirm that $\bar{k}=0.004>{\bar{k}}_{*}$ drives the system to $E_{0}^{*}$, whereas sub-threshold isolation $\left(\bar{k}=0.001\right)$ fails to curb transmission.

Our numerical simulations in the present section lead to two key observations based on threshold and upper bound comparisons:

1. The isolation threshold ${\bar{k}}_{*}$ is significantly lower than the protection threshold ${\bar{h}}_{*}$ $\left({\bar{k}}_{*}\ll {\bar{h}}_{*}\right)$. This implies that isolating the infected population can be implemented with a much lower intensity than protecting the susceptible individuals, while still being potentially more effective.
2. The isolation upper bound $B_{\text{up}}\left(\bar{h}=0\right)$ is significantly higher than the protection upper bound $B_{\text{up}}\left(\bar{k}=0,\;\bar{\ell }=h_{\max}-\bar{h}\right)$:

$$B_{\text{up}}\left(\bar{k}=0,\;\bar{\ell }=h_{\max}-\bar{h}\right)\ll B_{\text{up}}\left(\bar{h}=0\right).$$

This suggests that isolation, even at a lower intensity, may be more effective at controlling the spread of disease, even in more challenging scenarios. For instance, in the case where

$$B_{\text{up}}\left(\bar{k}=0,\;\bar{\ell }=h_{\max}-\bar{h}\right)<R_{0}<B_{\text{up}}\left(\bar{h}=0\right),$$

protection alone is insufficient to control the epidemic, whereas isolation may succeed.

These findings suggest that isolating infected individuals could be more effective than protecting susceptible ones in achieving epidemic control. However, this preliminary conclusion requires further investigation, as it depends on specific model assumptions, particularly the constraint $\bar{\ell }=h_{\max}-\bar{h}$, and may change when time-dependent control measures *h*(*t*), *ℓ*(*t*), and *k*(*t*) are considered. This will be explored in the following sections.

### 4.2 Optimal control formulation with time-dependent protection and isolation

In this subsection, we analyze System (2) with time-varying controls *h*(*t*), *ℓ*(*t*), and *k*(*t*) over the time interval [0, *τ*], where *τ* > 0. We then formulate an optimal control problem aimed at minimizing the number of infected individuals in System (4) over this interval. Next, we establish the existence and uniqueness of the optimal controls (*h*∗, *ℓ*∗, *k*∗), and we provide their explicit expressions. At the end of this subsection, we compute the time-varying reproduction number $R_{c,0}\left(t\right)$ as a function of the optimal controls and discuss how these controls influence the dynamics of System (2), from the perspective of $R_{c,0}\left(t\right)$.

In order to formulate the optimal control problem for our system (2), we define the admissible control set as follows:

$$U_{ad}≔\left\{\left(h\left(\cdot \right),\ell \left(\cdot \right),k\left(\cdot \right)\right)|\;\;\;\;\;\;\begin{array}{ll} & h,\ell ,k\in PC\left(\left[0,\tau \right];R^{+}\right),\\ & h_{\min}\leq h\left(t\right)\leq h_{\max},\\ & k_{\min}\leq k\left(t\right)\leq k_{\max},\\ & \ell_{\min}\leq \ell \left(t\right)\leq \ell_{\max} \end{array}\right\},$$

where $PC\left(\left[0,\tau \right];R^{+}\right)$ is the space of piecewise continuous functions from [0, *τ*] to $R^{+}$.

For any fixed $\left(h,\ell ,k\right)\in U_{ad}$, the second part of System (2) is locally integrable with respect to *t* ∈ [0, *τ*] and locally Lipschitz continuous with respect to (*S*,*I*,*P*,*Q*)^*T*^. Therefore, given positive initial conditions, System (2) has a unique, bounded, and positive solution (*S*,*I*,*P*,*Q*)^*T*^ ∈ Ω, with Ω given by (3). This ensures that the admissible set $A_{ad}$, defined below, is non-empty, closed and bounded:

$$A_{ad}≔\left\{y\left(\cdot \right)≔y\left(\cdot ,h,\ell ,k\right)|\;\begin{array}{c} y={\left(S,I,P,Q\right)}^{T}\in \Omega \text{is the unique solution of}\\ \text{System}\left(2\right)\text{corresponding to}\left(h,\ell ,k\right)\in U_{ad}\text{and}\\ y\text{satisfies System}\left(2\right)\text{for almost every}t\in \left[0,\tau \right] \end{array}\right\}.$$

We now define the cost functional $J_{\tau }$, which maps elements from $A_{ad}\times U_{ad}$ to $R^{+}$ as follows:

$$J_{\tau }\left(y,h,\ell ,k\right)=\int_{0}^{\tau }\underset{\text{Instantaneous Cost}}{\underbrace{\left[\omega_{i}I\left(r\right)+\frac{\omega_{h}}{2}h{\left(r\right)}^{2}+\frac{\omega_{l}}{2}\ell {\left(r\right)}^{2}+\frac{\omega_{k}}{2}k{\left(r\right)}^{2}\right]}}\;dr,$$

where $y={\left(S,I,P,Q\right)}^{T}\in A_{ad}$ is the unique solution of System (2) corresponding to controls $\left(h,\ell ,k\right)\in U_{ad}$. The term *ω*_*i*_*I*(*t*) represents the *epidemic cost* at time *t*, quantifying the weighted contribution of the infected population to the overall impact. Here, *ω*_*i*_ reflects the relative severity or societal importance attributed to the epidemic's burden. This term captures the cumulative effect of the epidemic on the population over the time interval [0, *τ*]. The term $\frac{\omega_{h}}{2}h{\left(t\right)}^{2}$ represents the *control cost* associated with the implementation of protection measures for susceptible individuals at time *t*, where *ω*_*h*_ denotes the gain of protection controller. The term $\frac{\omega_{l}}{2}\ell {\left(t\right)}^{2}$ represents the *control cost* associated with the release of the protected population back into the susceptible group at time *t*. Here, *ℓ*(*t*) denotes the control governing the rate of reintegration, while *ω*_*l*_ is a weighting parameter that reflects the cost, effort, or risk involved in this process. The term $\frac{\omega_{k}}{2}k{\left(t\right)}^{2}$ represents the *control cost* associated with the implementation of isolation measures for the infected population at time *t*, with *ω*_*k*_ denoting the gain of the isolation controller.

The choice of a quadratic structure for the control cost in the functional $J_{\tau }$ is not arbitrary, it is motivated by both theoretical and modeling considerations. From a mathematical viewpoint, this choice guarantees that the integrand of the cost functional is strictly convex with respect to the control variables (*h*, *ℓ*, *k*). This property ensures that the Hamiltonian associated with the optimal control problem is strictly convex in the controls, leading to a well-defined characterization of pointwise optimal controls via the Pontryagin Maximum Principle.

From a modeling perspective, the controls *h*(*t*), *ℓ*(*t*), and *k*(*t*) represent continuous intervention efforts, corresponding to protection, return, and isolation rates. These interventions are implemented progressively in the model rather than as binary decisions. Since the controls considered here represent continuously adjustable intervention rates rather than purely discrete policy decisions, a quadratic control cost structure was chosen instead of a linear one. Indeed, a linear cost would typically favor bang-bang-type strategies, which may be less appropriate for the modeling framework considered in this work. Moreover, the quadratic form captures increasing marginal costs: higher intervention levels require disproportionately greater resources. The resulting control strategies are therefore typically smoother and more suitable for practical implementation in our setting.

The objective is to minimize the cost functional $J_{\tau }$ by determining controls (*h*∗, *ℓ*∗, *k*∗) from $U_{ad}$ and the corresponding state variables $y^{*}={\left(S^{*},I^{*},P^{*},Q^{*}\right)}^{T}\in A_{ad}$. This leads to the following optimization problem:

(13) $$J_{\tau }\left(y^{*},h^{*},\ell^{*},k^{*}\right)=\min\left\{J_{\tau }\left(y,h,\ell ,k\right)|y\in A_{ad}\;\;\text{and}\;\;\left(h,\ell ,k\right)\in U_{ad}\right\}.$$

Definition 4.1 A solution (*y*∗, *h*∗, *ℓ*∗, *k*∗) of Problem (13) is called an optimal solution of System (2)*.* The control functions (*h*∗, *ℓ*∗, *k*∗) are called the optimal controls for System (2).

The following theorem establishes the existence of an optimal solution to the controlled System (2). For the sake of clarity, the proof is given in Appendix 6.

Theorem 4.1 *There exists at least one* $\left(y^{*},h^{*},\ell^{*},k^{*}\right)\in A_{ad}\times U_{ad}$ *solution of the Problem* (13).

#### 4.2.1 Characterization of the optimal controls (*h*∗, *ℓ*∗, *k*∗)

In this subsection, we establish the characterization of the optimal controls (*h*∗, *ℓ*∗, *k*∗). To achieve this, we employ the Pontryagin Maximum Principle (PMP) (Fleming & Rishel, 2012; Semenovich Pontryagin, 2018). Let *y* = (*S*,*I*,*P*,*Q*)^*T*^ be the state vector of System (2). The Hamiltonian function H, corresponding to System (2), is defined as

$$\begin{array}{lll} H\left(t\right)\;\; & ≔ & \;\;H\left(y\left(t\right),\lambda \left(t\right),h\left(t\right),\ell \left(t\right),k\left(t\right)\right),\\ & = & \underset{\text{Instantaneous Cost}}{\underbrace{\omega_{i}I\left(t\right)+\frac{\omega_{h}}{2}h{\left(t\right)}^{2}+\frac{\omega_{\ell }}{2}\ell {\left(t\right)}^{2}+\frac{\omega_{k}}{2}k{\left(t\right)}^{2}}}+\underset{\text{Dynamic Constraint}}{\underbrace{\lambda \left(t\right)y^{\prime }\left(t\right),}} \end{array}$$

where *λ*(*t*) = (*λ*_1_(*t*), …, *λ*_4_(*t*)) such that *λ*_*i*_(*t*) for *i* = 1, …, 4, are adjoint variables satisfying:

(14) $$\left\{\begin{array}{l} \lambda^{\prime }\left(t\right)=-\left(\frac{\partial H\left(t\right)}{\partial S\left(t\right)},\frac{\partial H\left(t\right)}{\partial I\left(t\right)},\frac{\partial H\left(t\right)}{\partial P\left(t\right)},\frac{\partial H\left(t\right)}{\partial Q\left(t\right)}\right),\;t\in \left[0,\tau \right],\\ \lambda_{i}\left(\tau \right)=0,\;i=1,\ldots ,4. \end{array}\right)$$

The following theorem characterizes the explicit form of the optimal controls (*h*∗, *ℓ*∗, *k*∗).

Theorem 4.2 *Let* (*h*∗, *ℓ*∗, *k*∗) *be the optimal controls of System* (2) *and* ${\left(S^{*},I^{*},P^{*},Q^{*}\right)}^{T}$ *be the corresponding state vector. Then,* (*h*∗(*t*), *ℓ*∗(*t*), *k*∗(*t*)) *are given by*

(15) $$h^{*}\left(t\right)=\max\left(\min\left(\frac{S^{*}\left(t\right)\left(\lambda_{1}\left(t\right)-\lambda_{3}\left(t\right)\right)}{\omega_{h}},h_{\max}\right),h_{\min}\right),$$

(16) $$\ell^{*}\left(t\right)=\max\left(\min\left(\frac{P^{*}\left(t\right)\left(\lambda_{3}\left(t\right)-\lambda_{1}\left(t\right)\right)}{\omega_{\ell }},\ell_{\max}\right),\ell_{\min}\right),$$

*and*

(17) $$k^{*}\left(t\right)=\max\left(\min\left(\frac{I^{*}\left(t\right)\left(\lambda_{2}\left(t\right)-\lambda_{4}\left(t\right)\right)}{\omega_{k}},k_{\max}\right),k_{\min}\right),$$

*for* *t* ∈ [0, *τ*]*, where* $\lambda \left(t\right)=\left(\lambda_{1}\left(t\right),\ldots ,\lambda_{4}\left(t\right)\right)$ *is the solution of the following adjoint system:*

$$\left\{\begin{array}{l} \lambda_{1}^{\prime }\left(t\right)=\left(\mu +h^{*}\left(t\right)+\beta I^{*}\left(t\right)+\bar{\beta }Q^{*}\left(t\right)\right)\lambda_{1}\left(t\right)-\left(\beta I^{*}\left(t\right)+\bar{\beta }Q^{*}\left(t\right)\right)\lambda_{2}\left(t\right)-h^{*}\left(t\right)\lambda_{3}\left(t\right),\\ \lambda_{2}^{\prime }\left(t\right)=\left(\mu +\alpha +k^{*}\left(t\right)-\beta S^{*}\left(t\right)\right)\lambda_{2}\left(t\right)+\beta S^{*}\left(t\right)\lambda_{1}\left(t\right)-k^{*}\left(t\right)\lambda_{4}\left(t\right)-\omega_{i},\\ \lambda_{3}^{\prime }\left(t\right)=\left(\mu +\ell^{*}\left(t\right)\right)\lambda_{3}\left(t\right)-\ell^{*}\left(t\right)\lambda_{1}\left(t\right),\\ \lambda_{4}^{\prime }\left(t\right)=\left(\mu +\bar{\alpha }\right)\lambda_{4}\left(t\right)+\bar{\beta }S^{*}\left(t\right)\lambda_{1}\left(t\right)-\bar{\beta }S^{*}\left(t\right)\lambda_{2}\left(t\right), \end{array}\right)$$

*for* *t* ∈ [0, *τ*] *with* *λ*_*i*_(*τ*) = 0*,* *i* = 1, …, 4.

proof For the sake of clarity, *the proof is provided in* Appendix 6. □

#### 4.2.2 Time-dependent controlled reproduction number $R_{c,0}\left(t\right)$

In this subsection, we analytically derive the time-dependent (also referred to as the instantaneous) controlled reproduction number, denoted by $R_{c,0}\left(t\right)$. This quantity, also known as the effective reproduction number, represents the average number of new infections generated by a single infected individual at time *t*, taking into account the impact of control strategies, namely, protection *h*(*t*), release *ℓ*(*t*), and isolation *k*(*t*). For further details on the concept of time-dependent, instantaneous, or time-varying reproduction numbers, we refer the reader to (Bettencourt & Ribeiro, 2008; Fraser, 2007; Gostic et al., 2020; Wallinga & Lipsitch, 2007).

In System (2), infectious individuals are divided into two groups that contribute directly to the generation of new infections through contact with susceptible individuals: (i) *Free infected* individuals *I*(*t*), who transmit the disease at rate *β*; and (ii) *Isolated infected* individuals *Q*(*t*), who are quarantined via the isolation control *k*(*t*) and transmit the disease at a reduced rate $\bar{\beta }$. The control measures affect the transmission dynamics by reducing the susceptible population through protection *h*(*t*), returning individuals from the protected class to the susceptible class via release *ℓ*(*t*), and isolating infected individuals through *k*(*t*).

We define $R_{c,0}\left(t\right)$ as the sum of the contributions from the free infected individuals, *I*(*t*), and the isolated infected individuals, *Q*(*t*):

$$R_{c,0}\left(t\right)=\underset{\text{Contribution from}I}{\underbrace{R_{c,I}\left(t\right)}}+\underset{\text{Contribution from}Q}{\underbrace{R_{c,Q}\left(t\right).}}$$

(i) Contribution from *I*(*t*): at any time *t*, the instantaneous removal rate of each individual in *I*(*t*) is *μ* + *α* + *k*(*t*), which corresponds to an instantaneous expected infectious period of $\frac{1}{\mu +\alpha +k\left(t\right)}$. During this instantaneous period, transmission occurs at rate *βS*(*t*). Therefore, the expected number of secondary infections generated by one infectious individual at time *t* is given by

$$R_{c,I}\left(t\right)=\frac{\beta S\left(t\right)}{\mu +\alpha +k\left(t\right)}.$$

(ii) Contribution from *Q*(*t*): each individual in *I*(*t*), with an instantaneous expected infectious period of $\frac{1}{\mu +\alpha +k\left(t\right)}$, is isolated at rate *k*(*t*). Once in *Q*(*t*), the individual remains infectious for an average duration of $\frac{1}{\mu +\bar{\alpha }}$ and transmits the disease at a reduced rate $\bar{\beta }S\left(t\right)$. Consequently, the expected number of new infections arising from the *Q*(*t*) class, per initially infected individual, is

$$R_{c,Q}\left(t\right)=\frac{k\left(t\right)}{\mu +\alpha +k\left(t\right)}\cdot \frac{\bar{\beta }S\left(t\right)}{\mu +\bar{\alpha }}.$$

Hence, the total controlled reproduction number is:

(18) $$R_{c,0}\left(t\right)=\underset{\text{Free infected}}{\underbrace{\frac{\beta S\left(t\right)}{\mu +\alpha +k\left(t\right)}}}+\underset{\text{Isolated infected}}{\underbrace{\frac{\bar{\beta }S\left(t\right)\cdot k\left(t\right)}{\left(\mu +\alpha +k\left(t\right)\right)\left(\mu +\bar{\alpha }\right)}}}.$$

As the expressions above depend on time through *k*(*t*) and *S*(*t*), the durations and reproduction numbers used should be regarded as instantaneous (expected) quantities.

Remark 4.1 When an epidemic occurs, the value of $R_{c,0}\left(t\right)$ indicates whether the epidemic is currently growing or declining*.* This quantity serves as a key indicator for assessing the effectiveness of control interventions over time*.* In particular, appropriate adjustments of the control functions can drive $R_{c,0}\left(t\right)$ below the critical threshold of 1. If there exists a first (or optimal) time *t*∗ ≥ 0 such that $R_{c,0}\left(t^{*}\right)<1$, the epidemic is expected to decline locally starting from *t*∗*.* Here, “the epidemic is expected to decline locally at time *t*∗” refers to the short-term behavior of the epidemic curve*.*

As a function of the basic reproduction number $R_{0}$, we can write,

$$\begin{array}{lll} R_{c,0}\left(t\right) & = & \left(\frac{\left(\mu +\alpha \right)S\left(t\right)}{\mu +\alpha +k\left(t\right)}+\frac{\bar{\beta }\left(\mu +\alpha \right)S\left(t\right)\cdot k\left(t\right)}{\beta \left(\mu +\bar{\alpha }\right)\left(\mu +\alpha +k\left(t\right)\right)}\right)\times R_{0},\\ & = & G\left(S\left(t\right),k\left(t\right)\right)\times R_{0}, \end{array}$$

where

$$G\left(S\left(t\right),k\left(t\right)\right)=\frac{\left(\mu +\alpha \right)S\left(t\right)}{\mu +\alpha +k\left(t\right)}+\frac{\bar{\beta }\left(\mu +\alpha \right)S\left(t\right)\cdot k\left(t\right)}{\beta \left(\mu +\bar{\alpha }\right)\left(\mu +\alpha +k\left(t\right)\right)}.$$

It is clear that *G* is an increasing function with respect to *S*(*t*). With respect to *k*(*t*), we compute:

$$\begin{array}{lll} \frac{\partial G\left(S\left(t\right),k\left(t\right)\right)}{\partial k\left(t\right)} & = & \frac{\left(\mu +\alpha \right)S\left(t\right)}{{\left(\mu +\alpha +k\left(t\right)\right)}^{2}}\left(-1+\frac{\bar{\beta }\beta \left(\mu +\bar{\alpha }\right)\left(\mu +\alpha +k\left(t\right)\right)-\bar{\beta }\beta k\left(t\right)\left(\mu +\bar{\alpha }\right)}{{\left(\beta \left(\mu +\bar{\alpha }\right)\right)}^{2}}\right),\\ & = & \frac{\left(\mu +\alpha \right)S\left(t\right)}{{\left(\mu +\alpha +k\left(t\right)\right)}^{2}}\left(-1+\frac{\bar{\beta }\beta \left(\mu +\bar{\alpha }\right)\left(\mu +\alpha \right)}{{\left(\beta \left(\mu +\bar{\alpha }\right)\right)}^{2}}\right),\\ & = & \frac{\left(\mu +\alpha \right)S\left(t\right)}{{\left(\mu +\alpha +k\left(t\right)\right)}^{2}}\left(-1+\frac{\bar{\beta }}{\beta }\cdot \frac{\mu +\alpha }{\mu +\bar{\alpha }}\right). \end{array}$$

Since $\bar{\beta }\leq \beta$ and $\alpha \leq \bar{\alpha }$, we conclude that:

$$\frac{\partial G\left(S\left(t\right),k\left(t\right)\right)}{\partial k\left(t\right)}\leq 0.$$

This implies that *G* is a decreasing function with respect to *k*(*t*).

Consequently, reducing the density of *S*(*t*) and increasing the intensity of *k*(*t*) contribute to decreasing $R_{c,0}\left(t\right)$ below the critical threshold of 1. Note that,

0≤G(S(t),k(t))≤1,for allt∈[0,τ].

The density of *S*(*t*) is influenced by the protection control *h*(*t*) and the release control *ℓ*(*t*). Therefore, we conclude that the controls *h*(*t*) and *ℓ*(*t*) affect the dynamics of $R_{c,0}\left(t\right)$ indirectly, while the control *k*(*t*) has a direct impact.

Fig. 7 illustrates the behaviour of the reproduction number $R_{c,0}\left(\cdot \right)$ given by (18), as a function of the isolation control *k*(⋅) and the density of the susceptible population *S*(⋅) at a fixed time *t* = *t*∗ ≥ 0. As shown, there exist threshold values for both the isolation intensity and the density of susceptible individuals, beyond which the condition $R_{c,0}\left(t^{*}\right)<1$ can be achieved, allowing the epidemic to start declining.

**Fig. 7:**
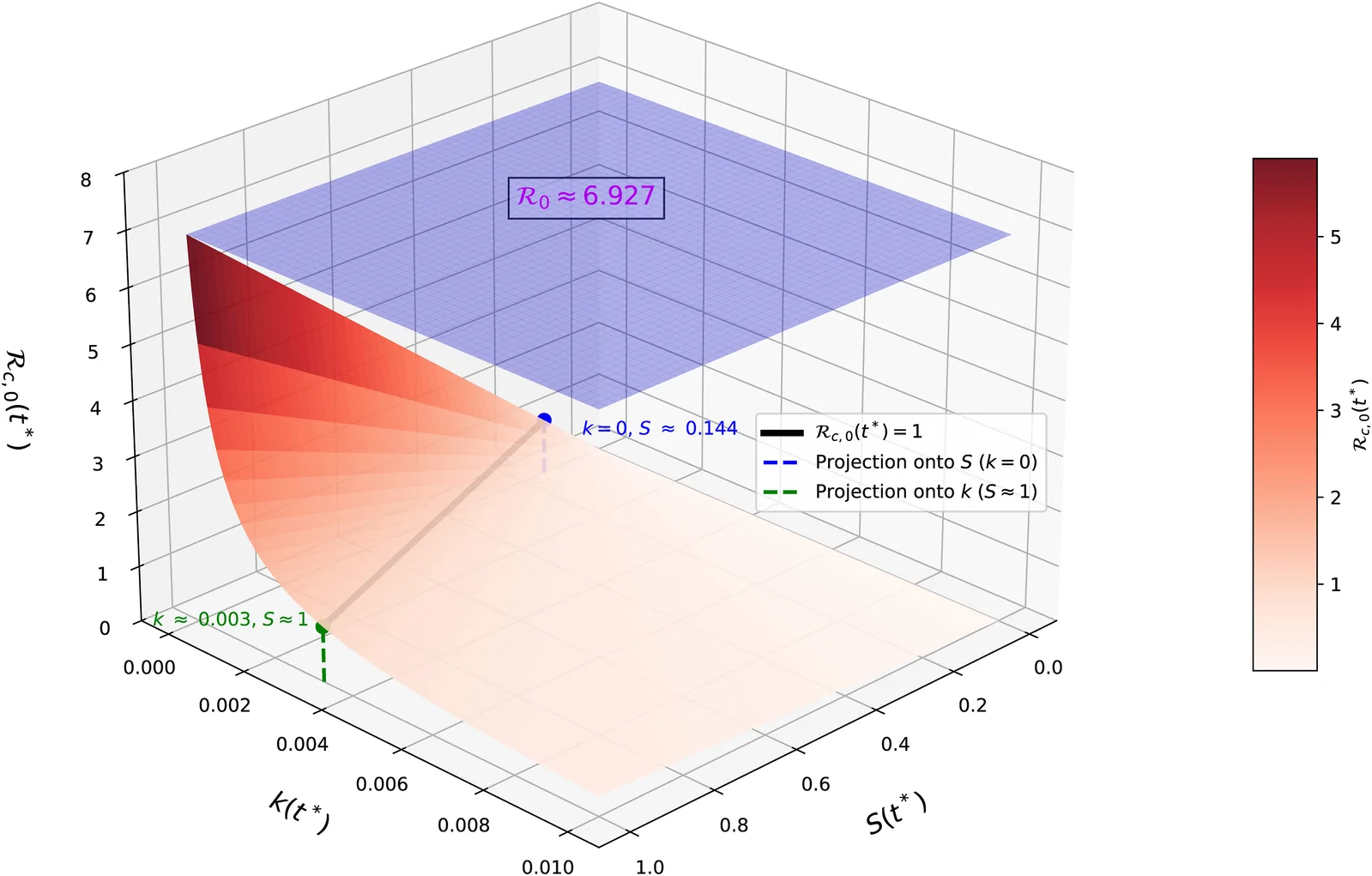
Dynamics of the reproduction number $R_{c,0}\left(\cdot \right)$ as a function of the susceptible population *S*(⋅) and the isolation control *k*(⋅) at a specified time *t*∗ ≥ 0. The parameters are set as follows: *k*_min_ = 0, *k*_max_ = 0.01, *μ* = 5 × 10^−5^, *β* = 3.81 × 10^−3^, $\bar{\beta }=2.8\times 10^{-8}$, *α* = 5 × 10^−4^, and $\bar{\alpha }=1.9\times 10^{-3}$. In this configuration, the basic reproduction number is $R_{0}\approx 6.927$.

Specifically, consider a moment *t*∗ when the density of the susceptible population is at its maximum and an infection is present (as confirmed by $R_{0}\approx 6.927>1$). To ensure that $R_{c,0}\left(t^{*}\right)<1$, the isolation control at time *t*∗ must exceed a critical threshold, *i.e.*, *k*(*t*∗) > *k*_∗_ = 0.003. This condition is sufficient to initiate a decline in the epidemic from time *t*∗. Conversely, if there is no isolation at time *t*∗, *i.e.*, *k*(*t*∗) = 0, then the density of susceptible individuals must be strictly less than a threshold *S*_∗_ ≈ 0.144 to satisfy $R_{c,0}\left(t^{*}\right)<1$. In this case, protecting susceptible individuals through an optimal protection control *h* and liberating them using an optimal return control *ℓ* becomes essential. These controls must also exceed certain thresholds, denoted *h*_∗_ and *ℓ*_∗_. However, since $R_{c,0}\left(t\right)$ does not depend directly on *h*(*t*) and *ℓ*(*t*), it is difficult at this stage to determine even approximate values for these thresholds. Overall, there exists a threshold condition that must be satisfied for the protection of susceptible and isolation of infected individuals to effectively control the spread of the disease, *i.e.*, to ensure that $R_{c,0}\left(t\right)<1$. This threshold corresponds to the projection onto the *k*(*t*)- and *S*(*t*)-axes of the contour line defined by $R_{c,0}\left(t\right)\left(k\left(t\right),S\left(t\right)\right)=1$.

### 4.3 Numerical simulations and discussion

In this subsection, we plot and analyze the dynamics of System (2). We set *β* = 3.81 × 10^−3^, *α* = 5 × 10^−4^ and *μ* = 5 × 10^−5^ corresponding to a high infection level $\left(R_{0}\approx 6.93>1\right)$. The objective is to control the epidemic's progression over a fixed time horizon of *τ* = 30 days, with the aim of achieving a rapid decline. If this duration proves insufficient, *τ* can be extended; however, determining the smallest possible value of *τ* that still ensures epidemic control is also of interest.

To achieve this, we aim to bring $R_{c,0}\left(t=t^{*}\right)<1$ as early as possible (*i.e.*, at the smallest time *t*∗, which represents the onset of epidemic decline), by employing control strategies focused on protecting susceptible individuals and isolating infected individuals, in a comparative framework. Accordingly, three distinct scenarios are investigated: (i) combined protection and isolation controls, (ii) isolation of infected individuals only, and (iii) protection of susceptible individuals only. The baseline parameter set is provided in Table 1. For each scenario, any modifications in parameter values are explicitly indicated and discussed. The simulations were performed using the ⓒ Python Programming Toolbox (Pontryagin Gradient Method (PGM)).

**Table 1:** Model parameters and their corresponding values.

Parameter	Value
*β*	3.81 × 10^−3^
$\bar{\beta }$	2.80 × 10^−8^
$\bar{\alpha }$	1.90 × 10^−3^
*τ*	30
*α*	5 × 10^−4^
*μ*	5 × 10^−5^
*h*_min_	0
*h*_max_	0.99
*ℓ*_min_	0.02
*ℓ*_max_	0.99
*k*_min_	0
*k*_max_	0.99

It is important to note that protection implicitly includes a return phase. Therefore, in the absence of protection, there is no associated return.

#### 4.3.1 Dynamics with combined protection and isolation

In this subsection, we analyze the implementation and impact of both protection and isolation controls on the dynamics of the epidemic. Fig. 8 illustrates the evolution of the isolation control *k*∗(*t*), protection control *h*∗(*t*), and return control *ℓ*∗(*t*), as well as the trajectories of the susceptible *S*∗(*t*), infected *I*∗(*t*), protected *P*∗(*t*), and isolated *Q*∗(*t*) populations. Three scenarios are considered for the initial densities of susceptible and infected populations to explore how control strategies can be applied under different epidemic conditions: $\left(S_{0},I_{0}\right)=\left\{\left(0.9,0.1\right),\left(0.7,0.3\right),\left(0.1,0.9\right)\right\}$, and *P*_0_ = *Q*_0_ = 0. Which is remarkable, in all scenarios, the density of the infected population decreases rapidly from the first day, reaching zero in approximately one week without exhibiting any peak. This indicates that the objective of minimizing the infected population is effectively achieved.

**Fig. 8:**
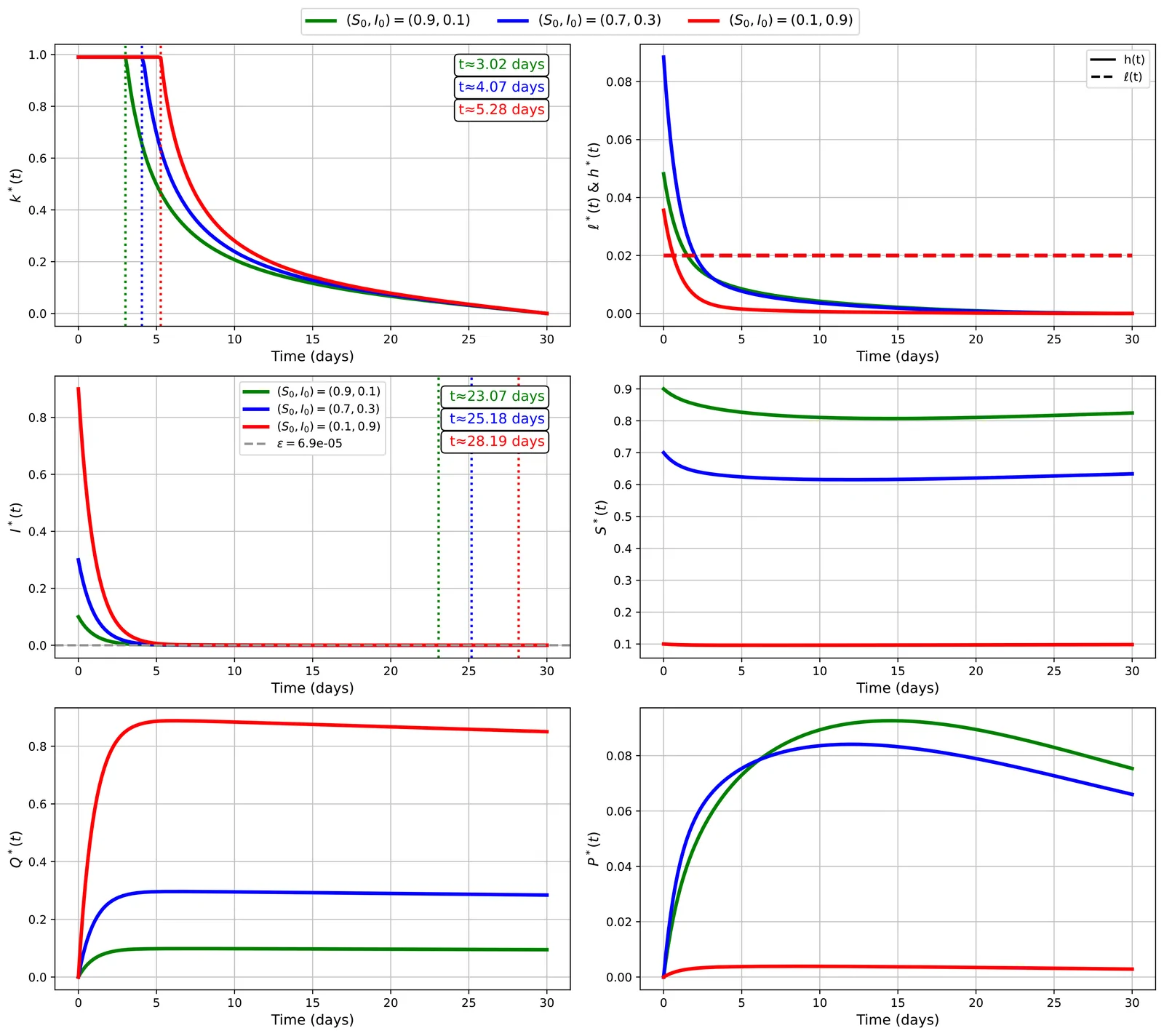
Temporal evolution of controls (isolation *k*∗(*t*), protection *h*∗(*t*), return *ℓ*∗(*t*)) and population states (susceptible *S*∗(*t*), infected *I*∗(*t*), protected *P*∗(*t*), isolated *Q*∗(*t*)) under combined protection/isolation measures over 30 days. Weights: infected density weight *w*_*i*_ = 10 and *w*_*k*_ = *w*_*h*_ = *w*_*ℓ*_ = 0.1. Three initial condition scenarios for susceptible and infected densities are shown: (*S*_0_, *I*_0_) ∈ {(0.9, 0.1), (0.7, 0.3), (0.1, 0.9)} with *P*_0_ = *Q*_0_ = 0. The vertical dotted lines in the subfigures of *I*∗(*t*) indicate the projected time needed to reduce the infection density below a small threshold *ɛ* (the objective). In the subfigures of *k*∗(*t*), they indicate the moments when the control changes from its maximum value to a lower one.

In the first scenario, (*S*_0_, *I*_0_) = (0.9, 0.1), corresponding to a low initial infection density, and represented by the green curves in Fig. 8, the isolation control *k*∗(*t*) is applied at maximum intensity but only for a short duration. The protection control *h*∗(*t*) starts at a low level and gradually decays to zero, a behavior that is expected, as the disease quickly vanishes due to the early, aggressive isolation effort. Meanwhile, the return control *ℓ*∗(*t*) remains at its minimum value throughout the containment period. In this case, relatively few individuals are isolated, whereas a larger fraction is protected to minimize contacts between susceptible and infected individuals, thereby achieving effective epidemic suppression. The high initial density of susceptible individuals justifies the large proportion of the population placed under protection, while the low initial density of infected individuals accounts for the limited number of isolated individuals.

In the second and third scenarios, (*S*_0_, *I*_0_) = (0.7, 0.3) and (*S*_0_, *I*_0_) = (0.1, 0.9), represented by the blue and red curves in Fig. 8, respectively, we observe that the duration of maximum-intensity isolation control increases with the initial infection density. This is intuitive: a higher initial infection density requires prolonged isolation to effectively suppress disease transmission. The protection control *h*∗(*t*) starts at a higher intensity in the second scenario than in the third, reflecting the intermediate initial densities of both susceptible and infected populations. As a result, the protected population density rises substantially in the second scenario. By contrast, the third scenario, characterized by a lower initial susceptible density, employs weaker protection, resulting in fewer protected individuals overall. In both cases, the infected population density *I*∗(*t*) rapidly declines to zero due to the aggressive early-stage isolation, which also explains the dynamics observed in the isolated population *Q*∗(*t*). As in the first scenario, the return control *ℓ*∗(*t*) remains at its minimum value throughout. The vertical dotted lines in the subfigures of *I*∗(*t*) indicate the projected time needed to reduce the infection density below a small threshold *ɛ* = 0.69 × 10^−4^ (the objective). In the subfigures of *k*∗(*t*), they indicate the moments when the control changes from its maximum value to a lower one.

In summary, our analysis shows that the combined control strategy effectively suppresses the epidemic in all scenarios, rapidly driving the infected population to zero without generating secondary peaks. The intensity and duration of the isolation control *k*∗(*t*) and protection control *h*∗(*t*) adapt to the initial conditions, with early, aggressive isolation playing a pivotal role. Higher initial infection densities necessitate prolonged maximum isolation, whereas protection measures scale with the size of the susceptible population. The return control *ℓ*∗(*t*) remains at its minimum value throughout the containment period. Overall, the strategy successfully minimizes transmission while optimizing the allocation of resources between protection and isolation interventions.

#### 4.3.2 Dynamics with isolation only

We consider the scenario where *h*(*t*) = 0, *ℓ*(*t*) = 0 and *P*(*t*) = 0 for all *t* ∈ [0, *τ*], meaning that isolating the infected population is the only control. Hence, System (2) is reduced to the following form, for *t* ∈ [0, *τ*]:

(19) $$\left\{\begin{array}{ll} S^{\prime }\left(t\right) & =\mu -\mu S\left(t\right)-\left(\beta I\left(t\right)+\bar{\beta }Q\left(t\right)\right)S\left(t\right),\\ I^{\prime }\left(t\right) & =-\mu I\left(t\right)-\alpha I\left(t\right)+\left(\beta I\left(t\right)+\bar{\beta }Q\left(t\right)\right)S\left(t\right)-k\left(t\right)I\left(t\right),\\ Q^{\prime }\left(t\right) & =-\mu Q\left(t\right)-\bar{\alpha }Q\left(t\right)+k\left(t\right)I\left(t\right). \end{array}\right)$$

Some additional mathematical analyses of this system are presented below for clarity and confidence. The isolation control *k*(*t*) is chosen from the admissible controls set defined by:

$$U_{ad}\left(Q\right)≔\left\{k\left(\cdot \right)|\;\begin{array}{ll} & k\text{is piecewise continuous on}\left[0,\tau \right],\\ & k_{\min}\leq k\left(t\right)\leq k_{\max} \end{array}\right\}.$$

We also define the non-empty, closed, and bounded admissible set $A_{ad}\left(Q\right)$ as:

$$A_{ad}\left(Q\right)≔\left\{y\left(\cdot \right)≔y\left(\cdot ,k\right)|\;\begin{array}{c} y={\left(S,I,Q\right)}^{T}\text{is the unique solution of System}\left(19\right)\\ \text{corresponding to}k\in U_{ad}\left(P\right),\text{satisfying}\left(19\right)\text{for}\\ \text{almost every}t\in \left[0,\tau \right]\text{with}0\leq S,I,Q\leq 1 \end{array}\right\}.$$

The cost functional $J_{\tau ,Q}:A_{ad}\left(Q\right)\times U_{ad}\left(Q\right)\to R^{+}$ is defined as:

$$J_{\tau ,Q}\left(y,k\right)=\int_{0}^{\tau }\underset{\text{Instantaneous Cost}}{\underbrace{\left[\omega_{i}I\left(r\right)+\frac{\omega_{k}}{2}k{\left(r\right)}^{2}\right]}}\;dr,$$

where $y={\left(S,I,Q\right)}^{T}\in A_{ad}\left(Q\right)$ is the unique solution of System (19) corresponding to the control $k\in U_{ad}\left(Q\right)$. By Theorem 6.1, and following a similar approach to the proof of Theorem 4.1, we show the existence of a pair $\left(y^{*},k^{*}\right)\in A_{ad}\left(Q\right)\times U_{ad}\left(Q\right)$ such that

$$J_{\tau ,Q}\left(y^{*},k^{*}\right)=\min\left\{J_{\tau ,Q}\left(y,k\right)|\left(y,k\right)\in A_{ad}\left(Q\right)\times U_{ad}\left(Q\right)\right\},$$

where $y^{*}={\left(S^{*},I^{*},Q^{*}\right)}^{T}$ and *k*∗ satisfy

$$k^{*}\left(t\right)=\max\left(\min\left(\frac{I^{*}\left(t\right)\left(\lambda_{2}\left(t\right)-\lambda_{3}\left(t\right)\right)}{\omega_{k}},k_{\max}\right),k_{\min}\right),$$

for *t* ∈ [0, *τ*], and *λ*(*t*) = (*λ*_1_(*t*), *λ*_2_(*t*), *λ*_3_(*t*)) is the solution of the following adjoint system:

$$\left\{\begin{array}{l} \lambda_{1}^{\prime }\left(t\right)=\left(\mu +\beta I^{*}\left(t\right)+\bar{\beta }Q^{*}\left(t\right)\right)\lambda_{1}\left(t\right)-\left(\beta I^{*}\left(t\right)+\bar{\beta }Q^{*}\left(t\right)\right)\lambda_{2}\left(t\right),\\ \lambda_{2}^{\prime }\left(t\right)=\left(\mu +\alpha +k^{*}\left(t\right)-\beta S^{*}\left(t\right)\right)\lambda_{2}\left(t\right)+\beta S^{*}\left(t\right)\lambda_{1}\left(t\right)-k^{*}\left(t\right)\lambda_{3}\left(t\right)-\omega_{i},\\ \lambda_{3}^{\prime }\left(t\right)=\left(\mu +\bar{\alpha }\right)\lambda_{3}\left(t\right)+\bar{\beta }S^{*}\left(t\right)\lambda_{1}\left(t\right)-\bar{\beta }S^{*}\left(t\right)\lambda_{2}\left(t\right), \end{array}\right)$$

for *t* ∈ [0, *τ*], with terminal condition *λ*_*i*_(*τ*) = 0 for *i* = 1, 2, 3.

Here, *k*∗(⋅) represents the optimal isolation control associated with System (19).

For the simulations in this subsection, we consider the same initial scenarios as in the case where both control strategies, protection and isolation, are implemented. The initial conditions are (*S*_0_, *I*_0_) ∈ {(0.9, 0.1), (0.7, 0.3), (0.1, 0.9)}, with *P*_0_ = *Q*_0_ = 0. To complement our discussion, we also include additional simulations illustrating the effects of variations in key model parameters.

Fig. 9 shows the evolution of the optimal isolation control *k*∗(*t*) together with the corresponding trajectories of the infected population *I*∗(*t*) and the isolated population *Q*∗(*t*). A zoomed view of the last 10 days highlights the behavior of the infected population density. The vertical lines in the subfigures of *I*∗(*t*) indicate the projected time needed to reduce the infection density below a small threshold *ɛ* (the horizontal dashed line in the zoomed subfigure of *I*∗(*t*)); and in the subfigure of *k*∗(*t*) it indicates the moments when the control changes from its maximum value to a lower one. As illustrated, the implementation of isolation control results in a rapid decline in the number of infected individuals across all scenarios. Overall, the control objective, to minimize the number of infected individuals as quickly as possible, is successfully achieved.

**Fig. 9:**
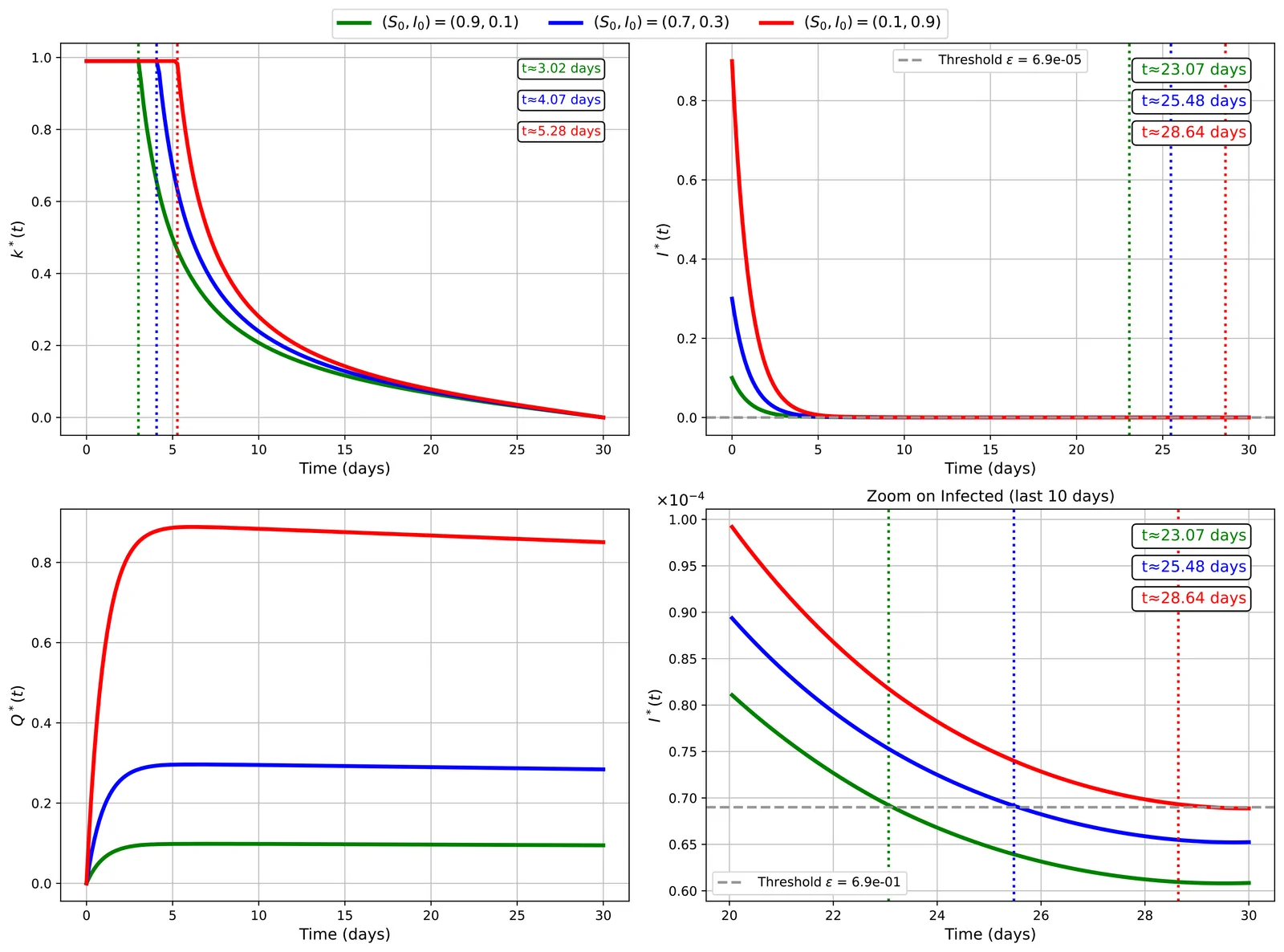
Temporal evolution of the isolation control *k*∗(*t*) and population states, susceptible (*S*∗(*t*)), infected (*I*∗(*t*)), and isolated (*Q*∗(*t*)), under isolation-only measure over 30 days. Weight parameters: *w*_*i*_ = 10 and *w*_*k*_ = 0.1. Initial conditions: (*S*_0_, *I*_0_) ∈ {(0.9, 0.1), (0.7, 0.3), (0.1, 0.9)} with *P*_0_ = *Q*_0_ = 0. The dotted vertical lines in the subfigures of *I*∗(*t*) indicate the projected time needed to reduce the infection density below a small threshold *ɛ*; and in the subfigure of *k*∗(*t*) it indicates the moments when the control changes from its maximum value to a lower one.

More precisely, the isolation control is initially applied at maximum intensity for a period whose duration depends on the initial density of infected individuals, *I*_0_. Higher initial infection levels require longer periods of aggressive isolation. After this initial phase, the control intensity gradually decreases as the number of infected individuals declines, which is expected as the epidemic wanes. The zoomed subfigure confirms that the infected population approaches zero during the final ten days of the month. The early application of intense isolation also explains the observed dynamics of the isolated population: higher initial infection levels correspond to larger numbers of isolated individuals.

Fig. 10, Fig. 11 present a sensitivity analysis of the optimal control *k*∗(*t*) and the corresponding infected population *I*∗(*t*) with respect to the key model parameters, namely *α*, *β*, $\bar{\alpha }$, and $\bar{\beta }$, as well as the cost weights *ω*_*i*_ and *ω*_*k*_.

**Fig. 10:**
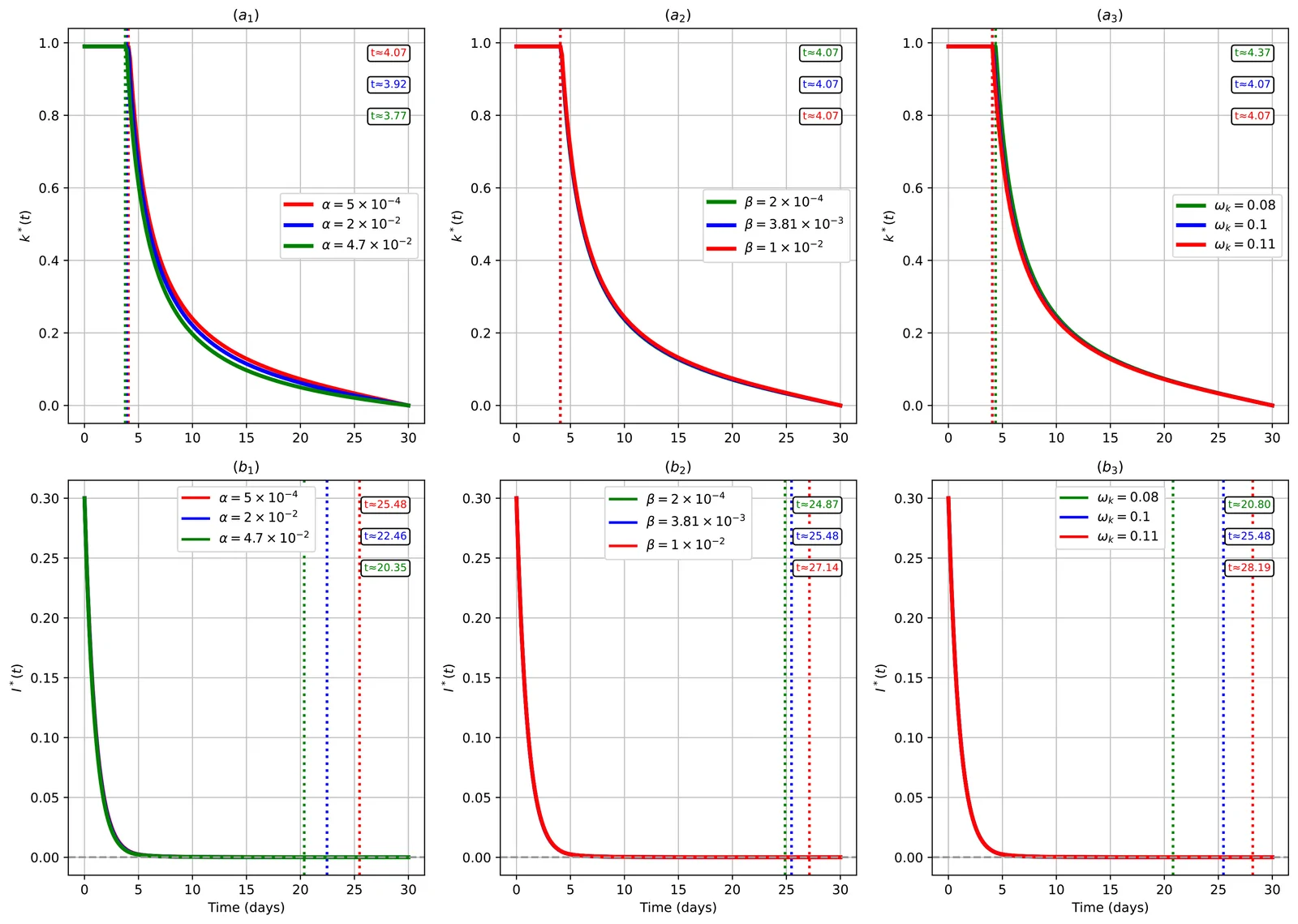
Sensitivity analysis of the optimal control *k*∗(*t*) and the corresponding infected population *I*∗(*t*) with respect to *α*, *β*, and *ω*_*k*_ in the absence of protection (*h*(*t*) = 0). The first row (*a*_1_)–(*a*_3_) illustrate the optimal isolation control *k*∗(*t*), while the second row (*b*_1_)–(*b*_3_) display the evolution of the infected population *I*∗(*t*). Each column corresponds to variations in a specific parameter: *α*, *β*, and *ω*_*k*_, respectively. Vertical dashed lines indicate critical switching times of the control (top panels) and the time required for *I*∗(*t*) to reach the prescribed threshold *ɛ* = 6.9 × 10^−5^ (bottom panels).

**Fig. 11:**
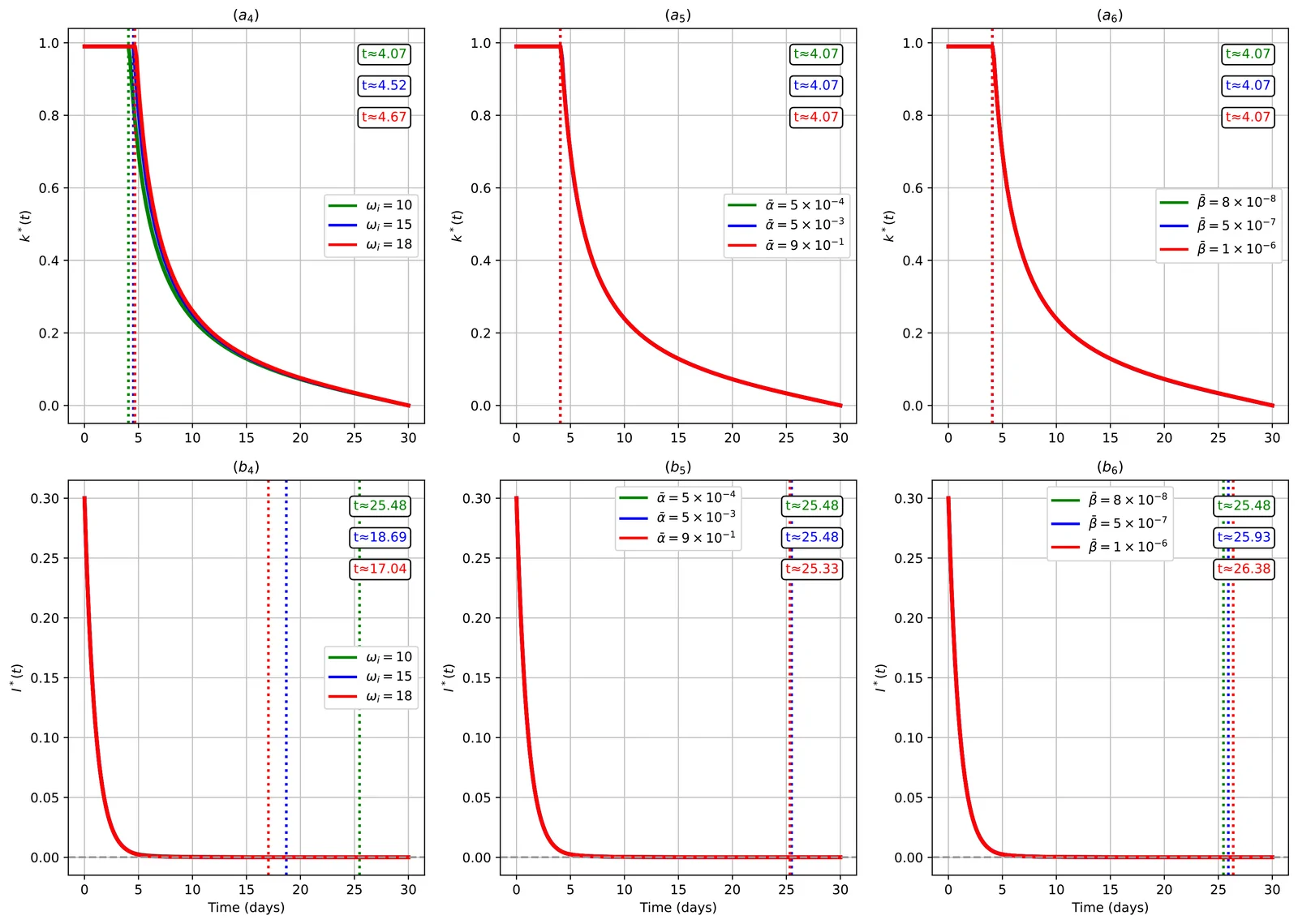
Sensitivity analysis of the optimal control *k*∗(*t*) and the corresponding infected population *I*∗(*t*) with respect to *ω*_*i*_, $\bar{\alpha }$, and $\bar{\beta }$ in the absence of protection (*h*(*t*) = 0). The first row (*a*_4_)–(*a*_6_) illustrate the optimal isolation control *k*∗(*t*), while the second row (*b*_4_)–(*b*_6_) display the evolution of the infected population *I*∗(*t*). Each column corresponds to variations in a specific parameter: *ω*_*i*_, $\bar{\alpha }$, and $\bar{\beta }$, respectively. Vertical dashed lines indicate critical switching times of the control (top panels) and the time required for *I*∗(*t*) to reach the prescribed threshold *ɛ* = 6.9 × 10^−5^ (bottom panels).

The simulations show that variations in these parameters mainly affect the switching times of the optimal control *k*∗(*t*), as well as the time required to reach the desired threshold. In particular, higher transmission rates (*β* or $\bar{\beta }$) or lower recovery rates (*α* or $\bar{\alpha }$) tend to increase the time needed to achieve the control objective. Conversely, lower transmission rates or higher recovery rates reduce this duration.

Regarding the cost weights, the time required to reach the desired threshold increases when the weight associated with the infected population (*ω*_*i*_) decreases or when the cost of the control (*e.g.*, *ω*_*k*_) increases.

Overall, the control objective is achieved within the prescribed time horizon *τ*. When necessary, this objective can still be attained by increasing the value of *τ*.

When compared to the case where protection and isolation controls are implemented simultaneously, isolation alone still proves to be an effective strategy for controlling the spread of infection, even when the initial density of infected individuals is high. The main difference lies in the time required to bring the epidemic under control. Under the combined strategy, the epidemic is generally contained more rapidly, which is intuitive: protection measures reduce the number of susceptible individuals exposed to infection, thereby preventing new cases and accelerating the decline of the infected population. However, since our objective does not explicitly optimize the final time, both strategies, isolating alone versus combined control, can be regarded as equally effective in achieving the desired epidemiological outcomes.

In summary, our results demonstrate that isolation control consistently induces a rapid decline in the number of infected individuals across all scenarios. The control is initially applied at maximum intensity, particularly in cases with higher initial infection levels, and then gradually decreases as the epidemic wanes.

#### 4.3.3 Dynamics with protection only

In this scenario, we assume that *k*(*t*) = 0 and *Q*(*t*) = 0. System (2) simplifies for *t* ∈ [0, *τ*], to:

(20) $$\left\{\begin{array}{ll} S^{\prime }\left(t\right) & =\mu -\mu S\left(t\right)-\beta I\left(t\right)S\left(t\right)-h\left(t\right)S\left(t\right)+\ell \left(t\right)P\left(t\right),\\ I^{\prime }\left(t\right) & =-\mu I\left(t\right)-\alpha I\left(t\right)+\beta I\left(t\right)S\left(t\right),\\ P^{\prime }\left(t\right) & =-\mu P\left(t\right)-\ell \left(t\right)P\left(t\right)+h\left(t\right)S\left(t\right). \end{array}\right)$$

Our objective remains to minimize the number of infected individuals in System (20) over the time interval [0, *τ*]. As in the previous subsection, we present some additional mathematical analysis below to clarify the specific context and avoid any confusion with the general results discussed in Subsection 4.2. The protection and the return controls are selected from the admissible set:

$$U_{ad}\left(P\right)≔\left\{\left(h\left(\cdot \right),\ell \left(\cdot \right)\right)|\;\begin{array}{l} h,\ell \text{are piecewise continuous on}\left[0,\tau \right],\\ h_{\min}\leq h\left(t\right)\leq h_{\max},\\ \ell_{\min}\leq \ell \left(t\right)\leq \ell_{\max} \end{array}\right\}.$$

We define the non-empty, closed, and bounded admissible set $A_{ad}\left(P\right)$ as follows:

$$A_{ad}\left(P\right)≔\left\{y\left(\cdot \right)≔y\left(\cdot ,h,\ell \right)|\;\begin{array}{c} y={\left(S,I,P\right)}^{T}\text{is the unique solution of System}\left(20\right)\\ \text{corresponding to}\left(h,\ell \right)\in U_{ad}\left(P\right),\text{satisfying}\\ \left(20\right)\text{for almost every}t\in \left[0,\tau \right]\text{with}\\ 0\leq S,I,P\leq 1 \end{array}\right\}.$$

The cost functional $J_{\tau ,P}:A_{ad}\left(P\right)\times U_{ad}\left(P\right)\to R^{+}$ is defined as:

$$J_{\tau ,P}\left(y,h,l\right)=\int_{0}^{\tau }\underset{\text{Instantaneous Cost}}{\underbrace{\left[\omega_{i}I\left(r\right)+\frac{\omega_{h}}{2}h{\left(r\right)}^{2}+\frac{\omega_{\ell }}{2}\ell {\left(r\right)}^{2}\right]}}\;dr,$$

where $y={\left(S,I,P\right)}^{T}\in A_{ad}\left(P\right)$ is the unique solution of System (20) corresponding to the control pair $\left(h,\ell \right)\in U_{ad}\left(P\right)$.

Following the same approach as in the proof of Theorem 4.1, there exists an optimal triple $\left(y^{*},h^{*},\ell^{*}\right)\in A_{ad}\left(P\right)\times U_{ad}\left(P\right)$ that minimizes the cost functional:

$$J_{\tau ,P}\left(y^{*},h^{*},\ell^{*}\right)=\min\left\{J_{\tau ,P}\left(y,h,l\right)|\left(y,h,l\right)\in A_{ad}\left(P\right)\times U_{ad}\left(P\right)\right\}.$$

Moreover, analogous to the proof of Theorem 4.2, we show that the optimal solution $y^{*}={\left(S^{*},I^{*},P^{*}\right)}^{T}$ with controls *h*∗ and *ℓ*∗ satisfies, for *t* ∈ [0, *τ*], the following optimality conditions:

$$h^{*}\left(t\right)=\max\left(\min\left(\frac{S^{*}\left(t\right)\left(\lambda_{1}\left(t\right)-\lambda_{3}\left(t\right)\right)}{\omega_{h}},h_{\max}\right),h_{\min}\right),$$

and

$$\ell^{*}\left(t\right)=\max\left(\min\left(\frac{P^{*}\left(t\right)\left(\lambda_{3}\left(t\right)-\lambda_{1}\left(t\right)\right)}{\omega_{\ell }},\ell_{\max}\right),\ell_{\min}\right),$$

where (*λ*_1_(*t*), *λ*_2_(*t*), *λ*_3_(*t*)) solves, for *t* ∈ [0, *τ*], the following adjoint system:

$$\left\{\begin{array}{l} \lambda_{1}^{\prime }\left(t\right)=\left(\mu +h^{*}\left(t\right)+\beta I^{*}\left(t\right)\right)\lambda_{1}\left(t\right)-\beta I^{*}\left(t\right)\lambda_{2}\left(t\right)-h^{*}\left(t\right)\lambda_{3}\left(t\right),\\ \lambda_{2}^{\prime }\left(t\right)=\left(\mu +\alpha -\beta S^{*}\left(t\right)\right)\lambda_{2}\left(t\right)+\beta S^{*}\left(t\right)\lambda_{1}\left(t\right)-\omega_{i},\\ \lambda_{3}^{\prime }\left(t\right)=\left(\mu +\ell^{*}\left(t\right)\right)\lambda_{3}\left(t\right)-\ell^{*}\left(t\right)\lambda_{1}\left(t\right), \end{array}\right)$$

with terminal conditions *λ*_*i*_(*τ*) = 0, *i* = 1, 2, 3.

The numerical simulations presented in this subsection illustrate the dynamics of System (20) when only the protection control is applied, that is, no isolation strategy is implemented over the considered time horizon. The initial population consists of 70% susceptible individuals and 30% infected individuals, corresponding to the initial condition (*S*_0_, *I*_0_) = (0.7, 0.3) with *P*_0_ = *Q*_0_ = 0. The objective is to evaluate how the epidemic evolves under different variations of key model parameters and cost weights. To this end, we analyze several distinct scenarios under the condition *τ* = 300. The value of *τ* is increased to illustrate that, even when it exceeds the fixed value given in Table 1, protection remains insufficient to control the epidemic (Fig. 12, Fig. 13), or it requires more time to become effective in the case of a high recovery rate (Fig. 14).

**Fig. 12:**
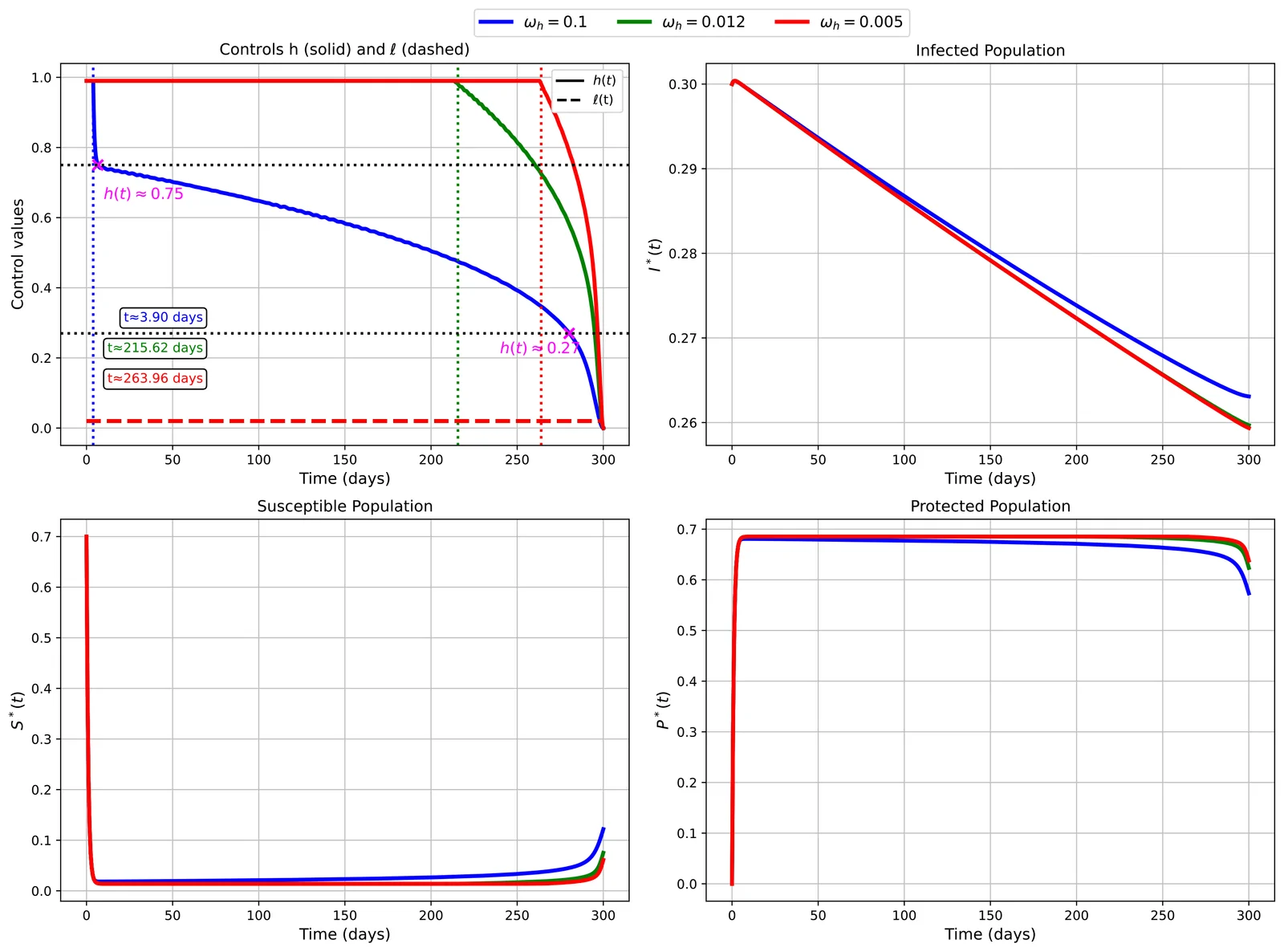
Temporal evolution of the optimal controls, protection *h*∗(*t*) and return *ℓ*∗(*t*), and population states: susceptible (*S*∗(*t*)), infected (*I*∗(*t*)), and protected (*P*∗(*t*)), under protection-only measures over *τ* = 300 days. Different protection weights *w*_*h*_ are tested, with fixed *w*_*i*_ = 10 and *ω*_*ℓ*_ = 0.1. The vertical lines in the subfigures of the controls indicate the moments when the control changes from its maximum value to a lower one. The initial condition is (*S*_0_, *I*_0_) = (0.7, 0.3) with *P*_0_ = *Q*_0_ = 0.

**Fig. 13:**
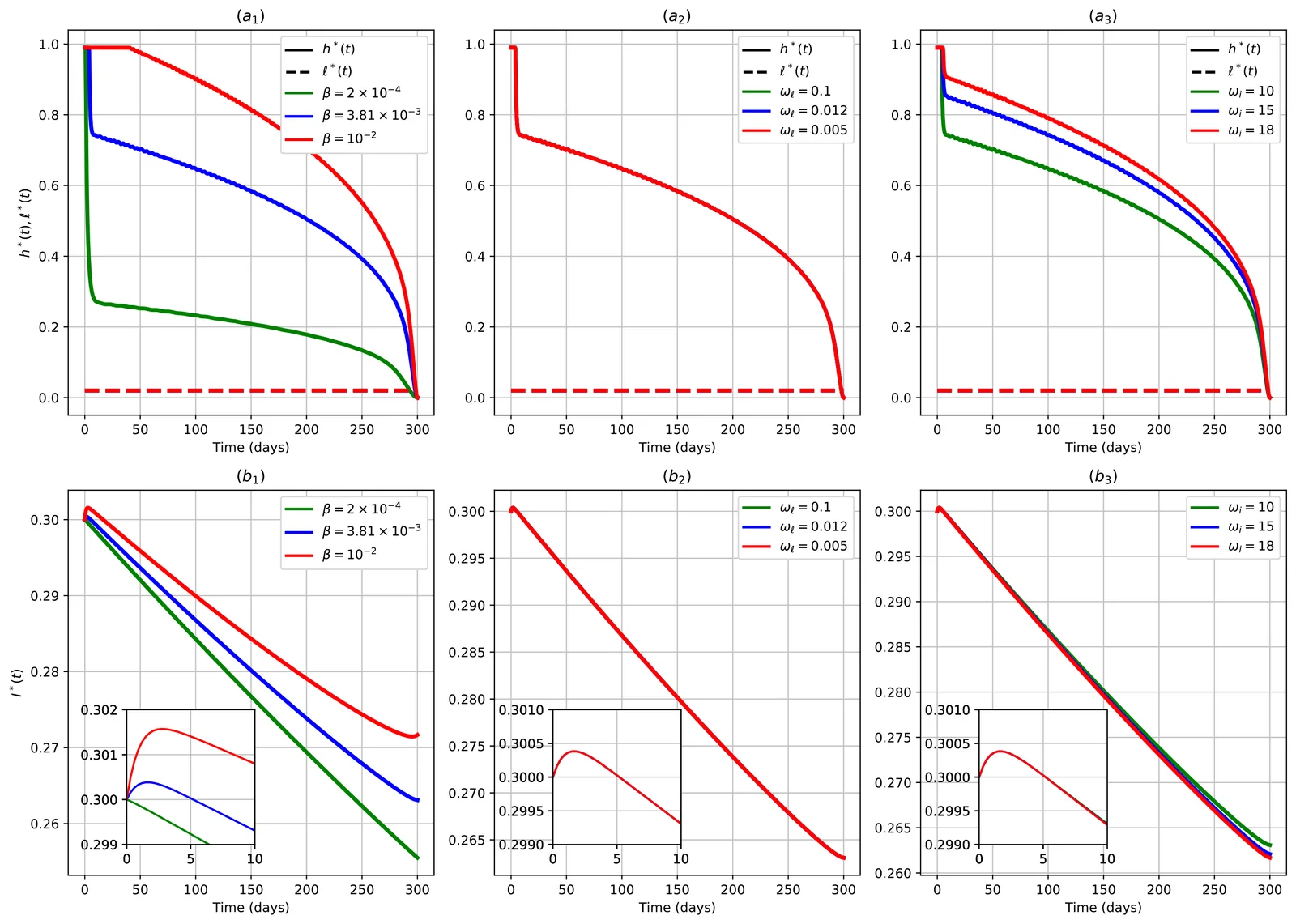
Sensitivity analysis of the optimal control *h*∗(*t*) and the corresponding infected population *I*∗(*t*) with respect to *β*, *ω*_*ℓ*_, and *ω*_*i*_ in the absence of isolation (*k*(*t*) = 0). The first row (*a*_1_)–(*a*_3_) illustrates the optimal protection control *h*∗(*t*), while the second row (*b*_1_)–(*b*_3_) displays the evolution of the infected population *I*∗(*t*). Each column corresponds to variations in a specific parameter: *β*, *ω*_*ℓ*_, and *ω*_*i*_, respectively. The horizontal dashed line indicates *ℓ*∗(*t*) over time. The simulations are performed over a time horizon of *τ* = 300 days.

**Fig. 14:**
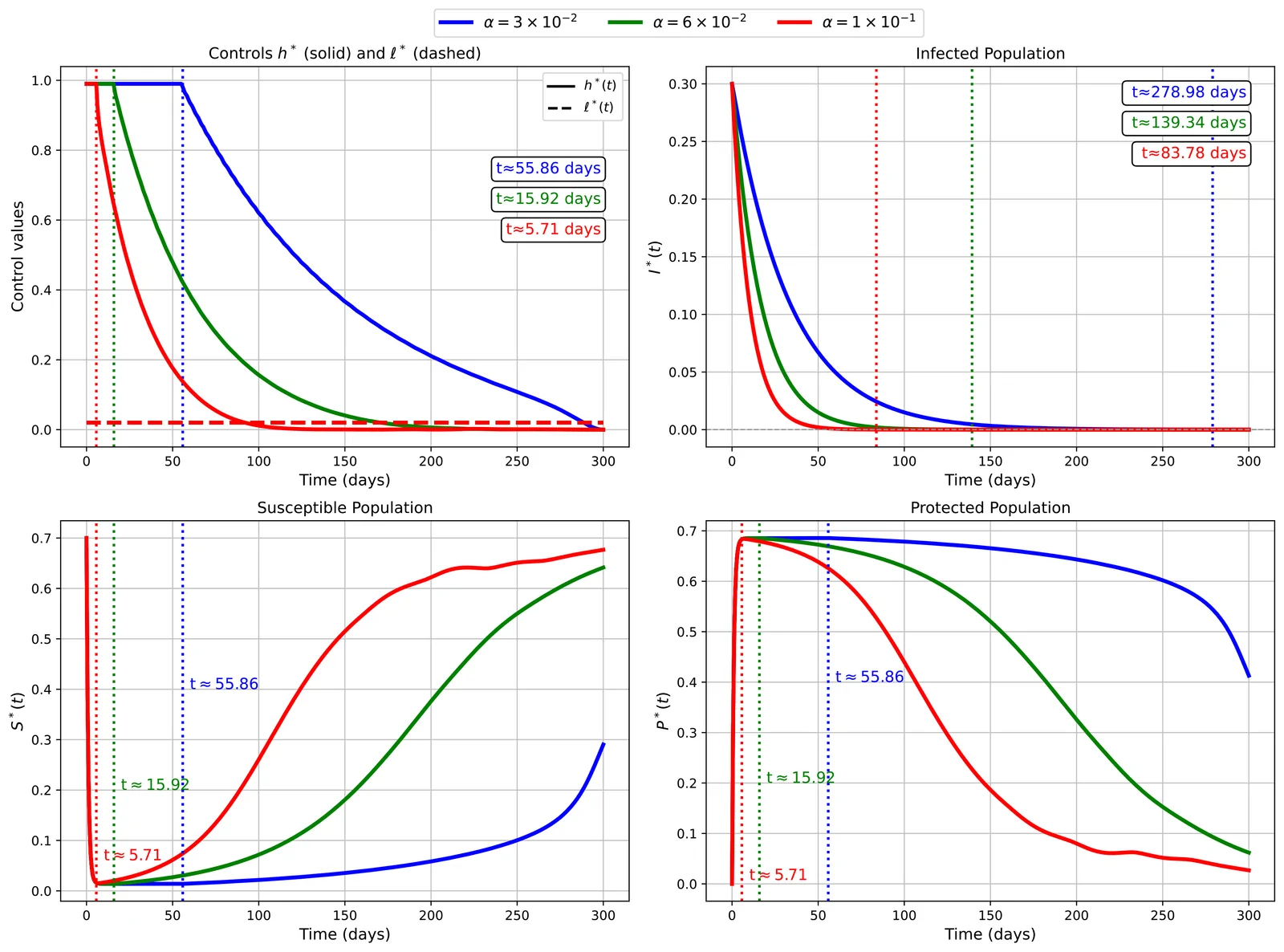
Temporal evolution of the optimal controls, protection *h*∗(*t*) and return *ℓ*∗(*t*), and population states: susceptible (*S*∗(*t*)), infected (*I*∗(*t*)), and protected (*P*∗(*t*)), under protection-only measures over *τ* = 300 days. Different recovery rates *α* are tested, with fixed *w*_*i*_ = 10, *w*_*h*_ = 0.001, and *ω*_*ℓ*_ = 0.1. The vertical lines in the subfigures of the controls, *S*∗(*t*) and *P*∗(*t*) indicate the moments when the control changes from its maximum value to a lower one. The vertical lines in the subfigures of *I*∗(*t*) indicate the projected time needed to stop disease transmission. Initial conditions: (*S*_0_, *I*_0_) = (0.7, 0.3) with *P*_0_ = *Q*_0_ = 0.

In the first case, we vary the weight parameter *ω*_*h*_, which represents the relative cost or priority associated with applying the protection control. We consider three values: *ω*_*h*_ = 0.1, corresponding to a high-cost scenario where the control is penalized more heavily; *ω*_*h*_ = 0.012, representing a moderate protection effort; and *ω*_*h*_ = 0.005, indicating a low-cost scenario that favors more aggressive protection. The other parameters are given in Table 1.

In the second case, we investigate how different values of the transmission rate (*β*) and the cost weights (*ω*_*ℓ*_ *& ω*_*i*_) affect the implementation of the protection and reintegration controls, as well as the dynamics of the infected population. We consider three different variations: (i) for the transmission rate, *β* = 1 × 10^−4^ (slow transmission), *β* = 3.81 × 10^−3^ (intermediate transmission), and *β* = 1 × 10^−2^ (fast transmission); (ii) for the reintegration cost weight, *ω*_*ℓ*_ = 0.1 (high-cost scenario), *ω*_*ℓ*_ = 0.012 (moderate cost), and *ω*_*ℓ*_ = 0.005 (low-cost scenario); and (iii) for the epidemic cost weight, *ω*_*i*_ = 10 (low epidemic cost), *ω*_*i*_ = 15 (moderate epidemic cost), and *ω*_*i*_ = 18 (high epidemic cost). In each case, the remaining parameters are given in Table 1.

In the third case, we explore the effect of different recovery rates *α*, which determine the average time an individual remains infected before recovery. We test three representative values: *α* = 3 × 10^−2^ (slow recovery), *α* = 6 × 10^−2^ (intermediate recovery), and *α* = 1 × 10^−1^ (fast recovery). These variations aim to capture how the natural course of the disease influences the effectiveness of protection strategies. We fixed *w*_*i*_ = 10, *w*_*h*_ = 0.001, and *ω*_*ℓ*_ = 0.1. The other parameters are given in Table 1.

Fig. 12 illustrates the dynamics of System (20) when only the protection control is applied, considering different weight parameter *w*_*h*_. For *w*_*h*_ = 0.1, we focus on the implications of a high-cost control scenario in managing the spread of the infection. As it can be seen, the protection control is implemented in four distinct phases. In the first phase, the control is applied at its maximum intensity (*h*∗(*t*) = *h*_max_) for approximately *t* ≈ 3.90 days. In the second phase, its intensity rapidly decreases from the maximum value to about *h*(*t*) ≈ 0.75. This is followed by a third phase, characterized by a gradual decline from *h*(*t*) ≈ 0.75 to *h*(*t*) ≈ 0.27. Finally, in the fourth phase, the control decreases more rapidly again. During the initial phase, the susceptible population is rapidly and fully protected, as shown in the third and fourth subfigures of Fig. 12. After a certain period, reintegration of the protected individuals into the susceptible population begins, but this occurs at a constant and minimal rate (*ℓ*∗(*t*) = *ℓ*_min_). Despite this cautious reintegration, the epidemic is not fully controlled within a time frame of approximately ten months. The density of the infected population decreases slowly, indicating that more time is required for the situation to stabilize. In summary, when the emphasis is placed on minimizing control costs, the level of protection achieved is insufficient to effectively contain the epidemic, even when reintegration occurs at a low intensity.

By reducing the relative cost to *w*_*h*_ = 0.012 and *w*_*h*_ = 0.005, we aim to increase investment in order to better control the spread of the epidemic in this scenario. In both cases, the protection control is applied at its maximum level, *h*∗(*t*) = *h*_max_, for the longest possible duration: it is maintained for *t* = 215.62 days when *w*_*h*_ = 0.012, and for *t* = 263.93 days when *w*_*h*_ = 0.005. This implies that lower relative costs allow for longer periods of maximum protection. With this aggressive measure, the susceptible population is fully protected to prevent any contact with infected individuals, thereby halting the emergence of new cases. The density of the infected population begins to decrease after a slight peak in the initial days, which is reasonable since some individuals may have already been infected due to prior contact before the protection measure took full effect. However, this decline in the infected population is slow and may require a long period to reach zero, even with strong protective actions. This is partly because protection is not applied on the infected class, and also due to the low recovery rate, which plays a significant role in the persistence of infection. In general, when the recovery rate *α* is low and protection control is not applied on infected individuals, such measures remain insufficient to fully eradicate the infection. Consequently, the epidemic requires a longer time to be controlled, implying prolonged suffering for the population, particularly if protection of susceptible involves lockdowns or other measures with severe economic and social consequences.

In summary, the analysis highlights that applying only the protection control is not sufficient to fully eliminate the epidemic, regardless of the cost parameter *w*_*h*_. A high-cost scenario (*w*_*h*_ = 0.1) results in limited and phased application of the control, which leads to slow epidemic mitigation. Conversely, lower-cost scenarios (*w*_*h*_ = 0.012 and *w*_*h*_ = 0.005) allow for prolonged maximum protection, reducing new infections more effectively. In all scenarios, the reintegration control (*i.e.*, the return control *ℓ*∗(*t*)) remains at its minimum value. However, due to the low recovery rate and the indirect effect of protection control on infected individuals, the epidemic persists for a long period, even when the duration *τ* of the control is extended beyond the value given in Table 1. Thus, while increasing protection efforts is beneficial, it remains insufficient on its own to achieve rapid epidemic control. Next, we address the following question: does a higher recovery rate enhance the effectiveness of protection control?

Fig. 13 illustrates the dynamics of the protection and reintegration controls, as well as the corresponding infected population, for different values of the transmission rate and the cost weights associated with the epidemic and reintegration. As the transmission rate increases, the duration during which the protection control is applied at its maximum level becomes longer. In general, as discussed previously, the protection control is implemented in four phases; however, these phases may be modified or reduced depending on the value of the transmission rate. The reintegration control remains at its minimum value in all cases.

Despite the implementation of these control strategies, the infected population decreases slowly, and more time is required to reach the desired threshold. Varying the weight of the reintegration cost does not significantly affect the results, which is consistent with the fact that the reintegration control remains always at its minimum.

On the other hand, varying the weight of the epidemic cost has a noticeable impact. For a low epidemic cost (*e.g.*, *ω*_*i*_ = 10), the protection control is applied less aggressively compared to moderate (*ω*_*i*_ = 15) or high (*ω*_*i*_ = 18) epidemic costs. This leads to higher infection levels and increases the time needed to achieve the control objective.

Overall, even under favorable conditions, such as lower transmission rates or higher epidemic costs, the protection control alone remains insufficient to effectively contain the spread of the infection. It requires a longer control horizon, which may result in higher economic costs and a larger number of infected individuals, due to the absence of isolation measures.

Fig. 14 illustrates the dynamics of System (20) considering different recovery rates. For *α* = 3 × 10^−2^, corresponding to a slow recovery rate, the disease persists for a longer duration before eventually vanishing. During this phase, it is crucial to maintain the protection control at its upper bound, *h*∗(*t*) = *h*_max_, for approximately two months (around *t* = 55.86 days). This strategy ensures that a substantial portion of the susceptible population is protected from the disease. The third and fourth subfigures of Fig. 14 clearly illustrate this effect: the density of the susceptible population rapidly declines to zero within approximately one week (*t* ≈ 5.71 days), while the protected population reaches its peak. This outcome is expected, as the disease remains active during the initial stages. Even with this aggressive protection strategy, which gradually decreases after *t* = 55.86 days, the disease persists for a long period before finally vanishing, approximately at *t* = 287.98 days, as shown in the second subfigure of Fig. 14.

For *α* = 6 × 10^−2^, Fig. 14 shows that the duration of the protection control is significantly reduced compared to the previous case, decreasing from approximately two months (*t* ≈ 55.86 days) to two weeks (*t* ≈ 15.92 days). The protection control remains at its maximum value (*h*∗(*t*) = *h*_max_) during this initial two-week period, ensuring the susceptible population is fully protected and does not come into contact with infected individuals. This strategy is justified, as the disease remains present during this phase, and it takes approximately two weeks for the epidemic to start declining, eventually disappearing around *t* ≈ 139.34 days. Once this critical period has passed (after *t* = 15.92 days), the protected population is gradually reintegrated into the susceptible group. This transition is clearly depicted by the green curve in Fig. 14, showing a decline in the protected population as individuals return to the susceptible class.

For *α* = 1 × 10^−1^, the situation becomes even more interesting. In this scenario, the duration of the protection control is further reduced to just one week (around *t* = 5.71 days). During this period, the protection control remains at its maximum value (*h*∗(*t*) = *h*_max_), effectively containing the spread of the disease. As illustrated by the red curve in Fig. 14, the susceptible population is fully protected during the first week, leading to a rapid increase in the density of the protected population. Meanwhile, the number of infected individuals declines more swiftly than in the previous cases, reaching zero in a short time. This sharp decline results from the high recovery rate *α* = 1 × 10^−1^ and the absence of new infections from the susceptible group, as they are fully protected during this critical period. After the first week, the protected population is completely reintegrated into the susceptible class, marking the end of the protection phase. Consequently, the epidemic stabilizes more rapidly, highlighting the effectiveness of short-term but intense protective measures in mitigating the outbreak.

From the analysis of these three cases with different recovery rates *α*, we observe that the duration and intensity of the protection control play a crucial role in mitigating the spread of the disease. For a low recovery rate (*α* = 3 × 10^−2^), the disease persists longer, requiring an extended application of the protection control at its maximum value (*h*∗(*t*) = *h*_max_). As the recovery rate increases (*α* = 6 × 10^−2^), the duration of maximum protection control decreases to about two weeks. For a high recovery rate (*α* = 0.1), the epidemic is controlled more rapidly, with the protection control maintained at its maximum for only one week before the protected population is reintegrated. However, it should be noted that even with a high recovery rate, relying solely on protection to control the epidemic requires more time compared to using isolation alone or combining both measures.

Overall, when recovery rates are low, prolonged protection is essential to compensate for the slow elimination of the disease. Conversely, higher recovery rates allow for shorter but more intensive protection measures to effectively mitigate the outbreak. In all scenarios, the reintegration control (*i.e.*, the return control *ℓ*∗(*t*)) remains at its minimum value, indicating that a cautious reintegration process is also essential.

The numerical simulations presented in Fig. 15 compare the evolution of the infected population and the time-varying reproduction number $R_{c,0}\left(t\right)$ over a one-month period (30 days). These results indicate that isolating infected individuals allows for a faster and more effective control of the spread of the disease. In contrast, when only the protection of susceptible individuals is implemented, the decline in the infected population begins after approximately 1.68 days and proceeds more slowly. On the other hand, with isolation measures, the number of infected individuals starts to decrease from the very first day and at a much faster rate. The dynamics of the time-varying reproduction number $R_{c,0}\left(t\right)$ further support these findings by clearly indicating the onset of disease decline in each scenario. In cases where both control measures are combined or only the isolation control is implemented, the simulations show that $R_{c,0}\left(t\right)$ converges to $R_{0}$ as time approaches *τ*, indicating that *S*(*τ*) ≈ 1. This confirms that the dynamics of the system begin to converge to the disease-free equilibrium, demonstrating the effectiveness of the implemented measures in accelerating the convergence of the system towards the disease-free state.

**Fig. 15:**
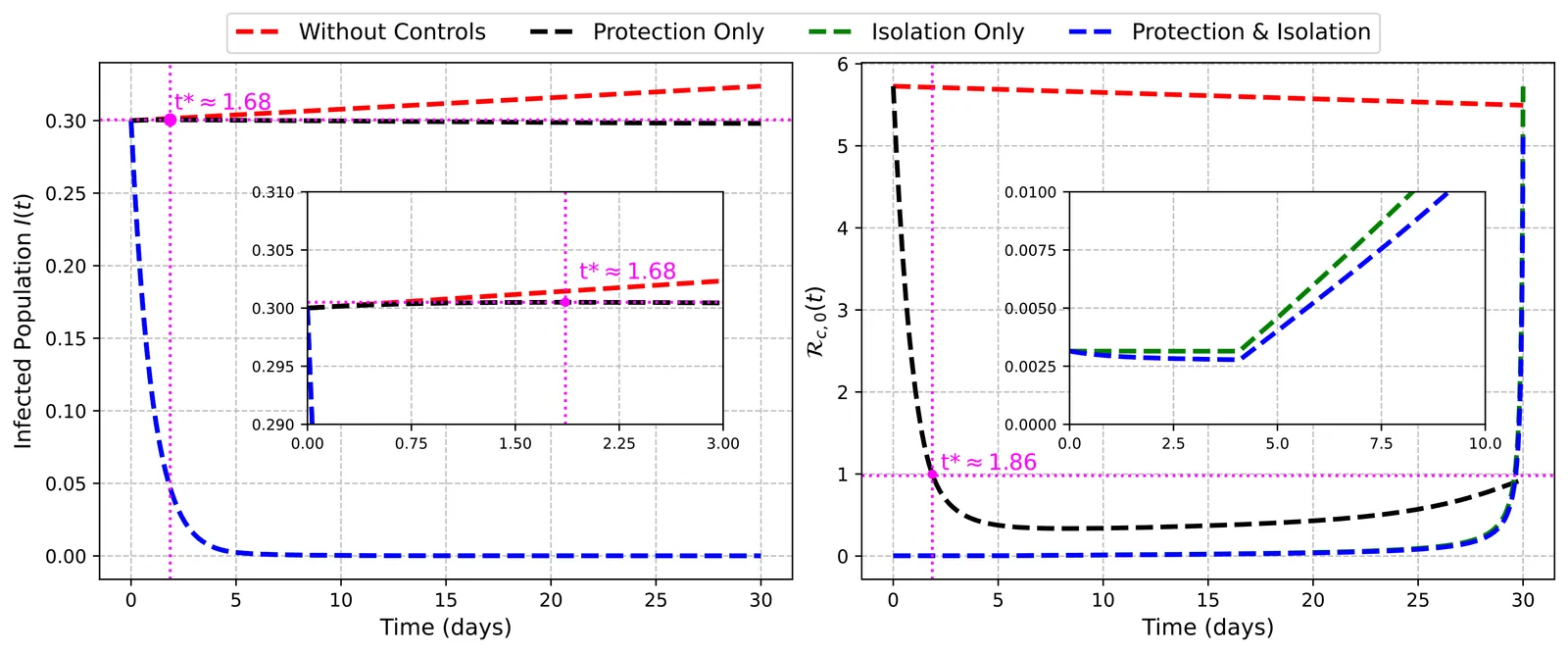
Comparison in the temporal evolution of the Infected population density and the time-varying reproduction number in four situations: (i) in the absence of controls, (ii) in the presence of both protection and isolation controls, (iii) in the presence of protection only, and (iv) in the presence of isolation only. The horizontal and vertical lines highlight the decline in the infected population when only the protection of susceptible individuals is implemented. Weights parameters are: *w*_*i*_ = 10 and *w*_*k*_ = *w*_*h*_ = *w*_*ℓ*_ = 0.1. Initial condition: (*S*_0_, *I*_0_) = (0.7, 0.3) with *P*_0_ = *Q*_0_ = 0.

The long-term outcomes illustrated in Fig. 16 reinforce these conclusions. After one year, the disease is completely eradicated when isolation is applied (*i.e.*, the proportion of infected individuals drops from 30% at the beginning to 0% after one year). In contrast, when only the protection of susceptible individuals is implemented, the extinction of the infected population takes considerably longer, potentially resulting in prolonged suffering and an increased risk for the entire population. It is important to highlight that, in contrast to the results reported in (Adimy et al., 2024), isolation emerged as the most effective strategy following the combination approach. This difference arises because, in (Adimy et al., 2024), the authors focus on long-term dynamics, whereas our study considers a short-term objective-specifically, controlling the spread of the epidemic within a limited time frame (parameter *τ*). For example, if we look at the simulation in Fig. 12 (for each *τ* = 30), it is clear that achieving long-term control requires applying protection at its maximum for as long as possible, which may not be feasible if protection corresponds to a lockdown, for example. Moreover, Fig. 14 suggests that effective protection requires a high recovery rate, which indirectly indicates that interventions targeting the infected population to increase the recovery rate may be necessary; in other words, control on the infected group must be applied when needed. In our case, it is an isolation control.

**Fig. 16:**
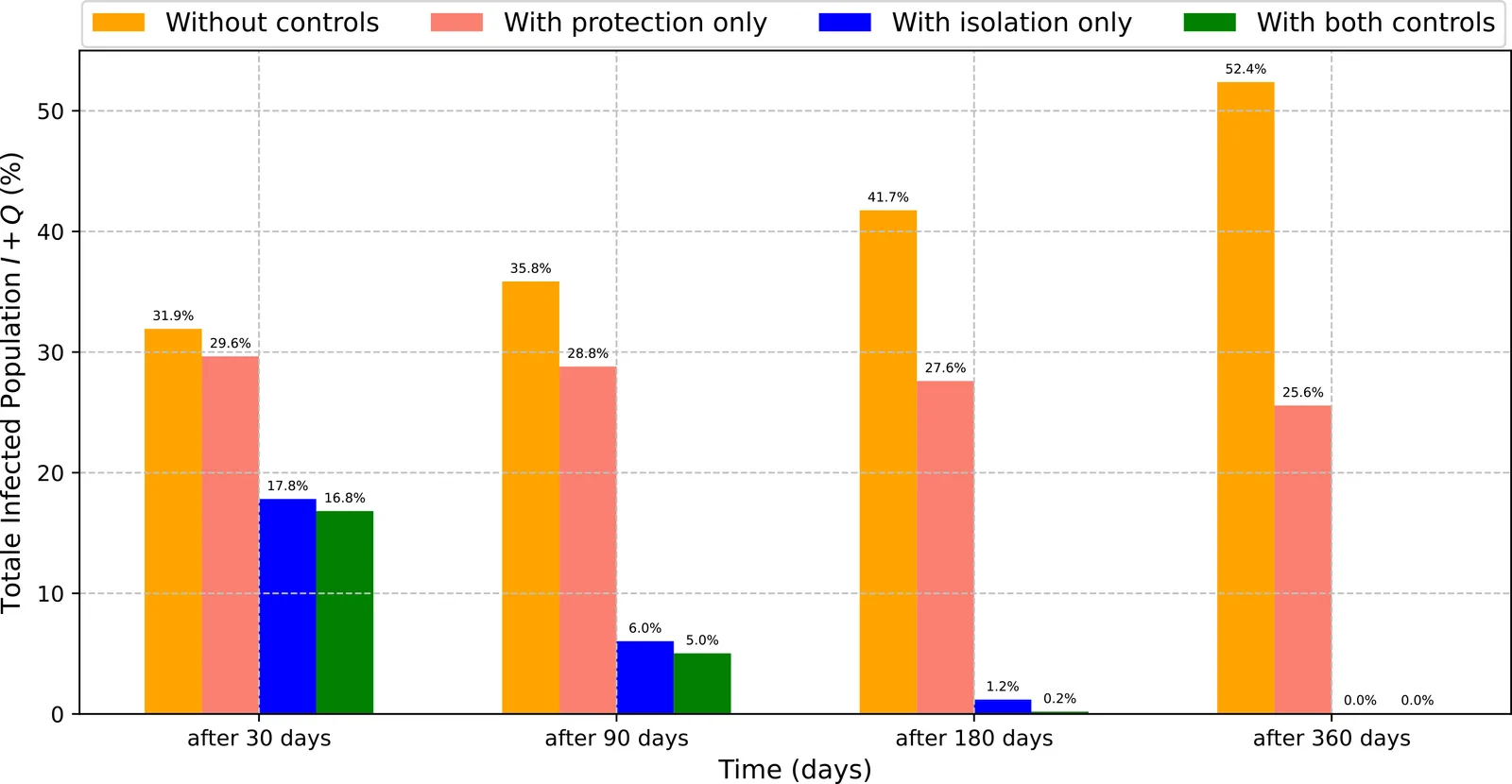
Comparison of infected population evolution over one year under different control strategies: no control, protection only, isolation only, and combined controls. Initial conditions: 30% infected and 70% susceptible individuals with *P*_0_ = *Q*_0_ = 0.

## 5 Conclusion

This study has shown that the combined implementation of protection and isolation policies provides the most effective strategy for controlling the epidemic. In this scenario, isolation is applied aggressively at the outset for a duration that depends on the initial proportion of infected individuals; higher prevalence requires longer periods of maximum isolation. Protection measures scale with the size of the susceptible population, while the return control remains at its minimum intensity throughout the containment period.

In the absence of protection but with isolation control in place, the strategy remains effective in curbing the spread of disease, even under conditions of high transmission and low recovery rates. Isolation is applied at maximum intensity initially and maintained for a duration comparable to the mean recovery time. A higher number of initial infections leads to a longer period of strict isolation. Despite the absence of protection for susceptible individuals, the number of infected individuals decreases rapidly, ultimately reaching zero within a reasonable time frame.

Conversely, when isolation is absent but protection is applied, the outcome depends strongly on the recovery rate. With a low recovery rate, the epidemic cannot be controlled within a reasonable period, leading to prolonged transmission and greater disease burden. However, when the recovery rate is high, protection alone is effective in limiting disease transmission. In this case, the required duration of protection varies: a shorter period suffices for a high recovery rate, whereas a longer protection phase is needed when recovery is slower.

In summary, this comparative study has highlighted the fundamental roles of protection and isolation in controlling infectious disease outbreaks within the classical SIR framework. The results clearly demonstrate that combining both strategies yields the most effective and rapid epidemic suppression under the considered conditions, while also revealing interesting situations in which the two strategies cannot be applied simultaneously. When only one measure can be implemented, isolating infected individuals is generally more effective than protecting the susceptible population, particularly under challenging epidemiological conditions. A comparative overview and policy recommendations for all examined scenarios are provided in Table 2.

**Table 2:** Comparative summary for all examined scenarios.

Strategy	Effectiveness	Best Use Case
Combination of Both	Most effective; achieves rapid and stable control.	General use, especially in high-risk or uncertain scenarios.
Isolation Alone	Highly effective, even under high transmission and low recovery rates.	When protection measures are impractical or unavailable.
Protection Alone	Conditionally effective; depends on recovery rate.	When recovery rates are high and isolation is difficult to enforce.

Nevertheless, the simplicity of the present model must be acknowledged. It does not account for key epidemiological features such as incubation periods, asymptomatic infections, or heterogeneous contact patterns, all of which could significantly influence epidemic dynamics and the relative effectiveness of interventions. Future research should extend this work by incorporating additional compartments, time delays, and population heterogeneities, thereby providing a more comprehensive and realistic assessment of optimal control strategies.

## CRediT authorship contribution statement

**Mohammed Elghandouri:** Writing – review & editing, Writing – original draft, Methodology, Investigation, Formal analysis. **Mostafa Adimy:** Writing – review & editing, Writing – original draft, Validation, Supervision, Methodology, Investigation, Formal analysis, Conceptualization. **Claudia Pio Ferreira:** Writing – review & editing, Writing – original draft, Validation, Investigation, Formal analysis, Conceptualization.

## Conflict of interest statement

The authors declare that they have no known competing financial interests or personal relationships that could have appeared to influence the work reported in this paper.

## Appendix

The following theorem will be used to prove the existence of the optimal controls. We recall it here for the reader's convenience. Theorem 6.1 *[11, Theorem 23.11] Consider the following optimal control problem defined on a fixed interval* [*a*, *b*]*:*

(21) $$\begin{array}{ll} & \text{Minimize}\;J\left(x,u\right)=l\left(x\left(a\right),x\left(b\right)\right)+\int_{a}^{b}\vartheta \left(t,x\left(t\right),u\left(t\right)\right)\;dt,\\ & \text{subject to}\;\left\{\begin{array}{l} x^{\prime }\left(t\right)=g_{0}\left(t,x\left(t\right)\right)+\overset{m}{\underset{j=1}{\sum }}g_{j}\left(t,x\left(t\right)\right)u^{j}\left(t\right)\text{a.e.},\\ u\left(t\right)\in U\left(t\right)\text{a.e.},\left(t,x\left(t\right)\right)\in F,\forall \;t\in \left[a,b\right],\\ \left(x\left(a\right),x\left(b\right)\right)\in E, \end{array}\right) \end{array}$$

*where* *U* *is the controls set, assumed to be Lebesgue measurable. For each* *t* ∈ [*a*, *b*]*, the set* $U\left(t\right)\subseteq R^{d}$ *with* *d* ≥ 1*, while* $F\subset \left[a,b\right]\times R^{n}$ *and* $E\subset R^{n}\times R^{n}$*. Let* ‖ ⋅‖_*p*_ *denote the Euclidean norm in* $R^{p}$ *with* *p* ≥ 1*. Assume that the following conditions are satisfied:*

(a) *For each* *j* ∈ {1, …, *m*}*,* $g_{j}:\left(t,z\right)\in F\to g_{j}\left(t,z\right)\in R^{d}$ *is such that* *g*_*j*_(*t*, *z*) *is Lebesgue measurable in* *t**, continuous in* *z**, and has linear growth: there exists a constant* *M* > 0 *such that*

$$\left(t,z\right)\in F\;⟹\;\|g_{j}\left(t,z\right){\|}_{d}\leq M\left(1+\|z{\|}_{n}\right);$$

(b) *For almost every* *t* ∈ [*a*, *b*]*, the set* *U*(*t*) *is closed and convex.*
(c) *The sets* *E* *and* *F* *are closed, and* $l:R^{n}\times R^{n}\to R$ *is lower semicontinuous;*
(d) *The running cost* *ϑ*(*t*, *z*, *w*) *is Lebesgue measurable in* *t* *and* (*z*, *w*)*, and lower semicontinuous in* (*z*, *w*)*;* *ϑ*(*t*, *z*, ⋅) *is convex for each* (*t*, *z*) ∈ *F**; there is a constant* *λ*_0_ > 0 *such that*

$$\left(t,z\right)\in F,w_{t}\in U\left(t\right)⟹\vartheta \left(t,z,w_{t}\right)\geq \lambda_{0};$$

(e) *The projection* $\left\{\alpha \in R^{n}:\left(\alpha ,\delta \right)\in E\text{for some}\delta \in R^{n}\right\}$ *of* *E* *is bounded;*
(f) *For some* *r* > 1*, there exists* $v\left(\cdot \right)\in L^{r}\left(a,b\right)$ *such that, for almost every* *t**,*

$$w_{t}\in U\left(t\right)⟹\|w_{t}{\|}_{d}\leq v\left(t\right).$$

*Then, if there is at least one admissible process* (*x*, *u*) *for which* *J*(*x*, *u*) *is finite, Problem* (21) *admits a solution.*

We use Theorem 6.1 for proving the existence of an optimal solution to System (2), which corresponds to a solution of Problem (13). Proof of Theorem 4.1 We need to rewrite the systems (2) and (13) in the form (21). Hence, by setting *a* = 0 and *b* = *τ*, the fixed interval is [0, *τ*]. Denote *x*(*t*) ≔ *y*(*t*) = (*S*(*t*),*I*(*t*),*P*(*t*),*Q*(*t*))^*T*^ and *u*(*t*)≔(*h*(*t*),*ℓ*(*t*),*k*(*t*))^*T*^. With these notations, the systems (2) and (13) can be rewritten in the form (21), where:

$$l=0,\;m=1,\;U=U_{ad},\;F=\left[0,\tau \right]\times A_{ad},\;E=\Omega \times \Omega \subset R^{4}\times R^{4},$$

$$\vartheta \left(t,x\left(t\right),u\left(t\right)\right)=\omega_{i}I\left(t\right)+\frac{\omega_{h}}{2}h{\left(t\right)}^{2}+\frac{\omega_{\ell }}{2}\ell {\left(t\right)}^{2}+\frac{\omega_{k}}{2}k{\left(t\right)}^{2},$$

$$g_{0}\left(t,x\left(t\right)\right)≔g_{0}\left(x\left(t\right)\right)=\left(\begin{array}{c} \mu -\left(\mu +\beta I\left(t\right)+\bar{\beta }Q\left(t\right)\right)S\left(t\right)\\ -\left(\mu +\alpha \right)I\left(t\right)+\left(\beta I\left(t\right)+\bar{\beta }Q\left(t\right)\right)S\left(t\right)\\ -\mu P\left(t\right)\\ -\left(\mu +\bar{\alpha }\right)Q\left(t\right) \end{array}\right),$$

and

$$g_{1}\left(t,x\left(t\right)\right)≔g_{1}\left(x\left(t\right)\right)=\left(\begin{array}{ccc} -S\left(t\right) & P\left(t\right) & 0\\ 0 & 0 & -I\left(t\right)\\ S\left(t\right) & -P\left(t\right) & 0\\ 0 & 0 & I\left(t\right) \end{array}\right),$$

for *t* ∈ [0, *τ*]. Under these notations, it can be easily verified that the conditions *(*a*)*-*(*f*)* of Theorem 6.1 are satisfied. Therefore, System (13) has at least one solution $\left(y^{*},h^{*},\ell^{*},k^{*}\right)\in A_{ad}\times U_{ad}$. □ Remark 6.1 **(Uniqueness via coercivity).** Let (*y*∗, *u*∗) = (*y*∗, *h*∗, *ℓ*∗, *k*∗) be a solution of Problem (13) with *u*∗ = (*h*∗, *ℓ*∗, *k*∗), and consider small variations *δu* = (*δh*, *δℓ*, *δk*)*.* The first variation of the system (2) is

$$\delta \dot{y}\left(t\right)=A\left(t\right)\;\delta y\left(t\right)+B\left(t\right)\;\delta u\left(t\right),\;\delta y\left(0\right)=0,$$

where

$$A\left(t\right)=\frac{\partial f}{\partial y}\left(y^{*}\left(t\right),u^{*}\left(t\right)\right)\;\text{and}\;B\left(t\right)=\frac{\partial f}{\partial u}\left(y^{*}\left(t\right),u^{*}\left(t\right)\right),$$

i.e.,

$$B\left(t\right)=\left(\begin{array}{ccc} -S^{*}\left(t\right) & P^{*}\left(t\right) & 0\\ 0 & 0 & -I^{*}\left(t\right)\\ S^{*}\left(t\right) & -P^{*}\left(t\right) & 0\\ 0 & 0 & I^{*}\left(t\right) \end{array}\right),$$

and

$$A\left(t\right)=\left(\begin{array}{cccc} -\left(\mu +\beta I^{*}\left(t\right)+\bar{\beta }Q^{*}\left(t\right)+h^{*}\left(t\right)\right) & -\beta S^{*}\left(t\right) & \ell^{*}\left(t\right) & -\bar{\beta }S^{*}\left(t\right)\\ \beta I^{*}\left(t\right)+\bar{\beta }Q^{*}\left(t\right) & -\left(\mu +\alpha +k^{*}\left(t\right)\right)+\beta S^{*}\left(t\right) & 0 & \bar{\beta }S^{*}\left(t\right)\\ h^{*}\left(t\right) & 0 & -\left(\mu +\ell^{*}\left(t\right)\right) & 0\\ 0 & k^{*}\left(t\right) & 0 & -\left(\mu +\bar{\alpha }\right) \end{array}\right).$$

*Let* Φ(*t*, *s*) be the state transition matrix of *A*(*t*):

$$\frac{d}{dt}\Phi \left(t,s\right)=A\left(t\right)\Phi \left(t,s\right),\;\Phi \left(s,s\right)=I_{4},\;\tau \geq t\geq s\geq 0.$$

Then the first variation of the state is

$$\delta y\left(t\right)=\int_{0}^{t}\Phi \left(t,s\right)B\left(s\right)\delta u\left(s\right)\;ds,\;\text{for}t\in \left[0,\tau \right].$$

The second variation of the cost functional reads

$$\delta^{2}J_{\tau }=\int_{0}^{\tau }\left[\omega_{h}{\left(\delta h\right)}^{2}+\omega_{\ell }{\left(\delta \ell \right)}^{2}+\omega_{k}{\left(\delta k\right)}^{2}\right]dt+\omega_{i}\int_{0}^{\tau }\delta^{2}I\left(t\right)dt,$$

where

$$\delta I\left(t\right)=e_{2}^{⊤}\delta y\left(t\right)=\int_{0}^{t}e_{2}^{⊤}\Phi \left(t,s\right)B\left(s\right)\delta u\left(s\right)ds.$$

Define the kernel

$$K\left(t,s\right)≔\Phi \left(t,s\right)B\left(s\right)\in R^{3\times 3}.$$

Since the states and controls are bounded, there exists a constant $C_{\tau }^{\prime }>0$ such that

$$\omega_{i}\int_{0}^{\tau }\delta^{2}I\left(t\right)dt\leq C_{\tau }^{\prime }\int_{0}^{\tau }\|\delta u\left(t\right){\|}^{2}dt.$$

Finally, the second variation satisfies

$$\delta^{2}J_{\tau }\geq \left(\min\left(\omega_{h},\omega_{\ell },\omega_{k}\right)-C_{\tau }^{\prime }\right)\int_{0}^{\tau }\|\delta u\left(t\right){\|}^{2}dt.$$

Hence, if

$$\min\left(\omega_{h},\omega_{\ell },\omega_{k}\right)>C_{\tau }^{\prime },$$

then $\delta^{2}J_{\tau }$ is coercive, and the solution (*y*∗, *h*∗, *ℓ*∗, *k*∗) is a strict global minimizer, ensuring global uniqueness. Proof of Theorem 4.2 Applying the Pontryagin Maximum Principle (PMP), (Fleming & Rishel, 2012; Semenovich Pontryagin, 2018), the optimal controls (*h*∗(*t*), *ℓ*∗(*t*), *k*∗(*t*)) must satisfy

$$\left(h^{*},\ell^{*},k^{*}\right)=\arg\min_{\left(h,\ell ,k\right)\in U_{ad}}H\left(t\right).$$

Using the optimality conditions, the optimal control functions (*h*∗, *ℓ*∗, *k*∗) can therefore be determined as follows:

$${\left(\frac{\partial H\left(t\right)}{\partial h\left(t\right)}\right|}_{h=h^{*},\;y=y^{*}}=0,\;{\left(\frac{\partial H\left(t\right)}{\partial \ell \left(t\right)}\right|}_{\ell =\ell^{*},\;y=y^{*}}=0,\;\text{and}\;{\left(\frac{\partial H\left(t\right)}{\partial k\left(t\right)}\right|}_{k=k^{*},\;y=y^{*}}=0.$$

Therefore, for *h*∗(*t*),

$$\begin{array}{lll} 0 & = & {\left(\frac{\partial H\left(t\right)}{\partial h\left(t\right)}\right|}_{h=h^{*},\;y=y^{*}}\\ & = & \omega_{h}h^{*}\left(t\right)-\lambda_{1}\left(t\right)S^{*}\left(t\right)+\lambda_{3}\left(t\right)S^{*}\left(t\right),\\ & = & \omega_{h}h^{*}\left(t\right)-S^{*}\left(t\right)\left(\lambda_{1}\left(t\right)-\lambda_{3}\left(t\right)\right). \end{array}$$

This implies that,

$$h^{*}\left(t\right)=\frac{S^{*}\left(t\right)\left(\lambda_{1}\left(t\right)-\lambda_{3}\left(t\right)\right)}{\omega_{h}}.$$

For *l*∗(*t*),

$$\begin{array}{lll} 0 & = & {\left(\frac{\partial H\left(t\right)}{\partial \ell \left(t\right)}\right|}_{\ell =\ell^{*},\;y=y^{*}}\\ & = & \omega_{\ell }\ell^{*}\left(t\right)+\lambda_{1}\left(t\right)P^{*}\left(t\right)-\lambda_{3}\left(t\right)P^{*}\left(t\right),\\ & = & \omega_{\ell }\ell^{*}\left(t\right)-P^{*}\left(t\right)\left(\lambda_{3}\left(t\right)-\lambda_{1}\left(t\right)\right). \end{array}$$

Then,

$$\ell^{*}\left(t\right)=\frac{P^{*}\left(t\right)\left(\lambda_{3}\left(t\right)-\lambda_{1}\left(t\right)\right)}{\omega_{\ell }}.$$

Similarly, for *k*∗(*t*),

$$\begin{array}{lll} 0 & = & {\left(\frac{\partial H\left(t\right)}{\partial k\left(t\right)}\right|}_{k=k^{*},\;y=y^{*}}\\ & = & \omega_{k}k^{*}\left(t\right)-\lambda_{2}\left(t\right)I^{*}\left(t\right)+\lambda_{4}\left(t\right)I^{*}\left(t\right),\\ & = & \omega_{k}k^{*}\left(t\right)-I^{*}\left(t\right)\left(\lambda_{2}\left(t\right)-\lambda_{4}\left(t\right)\right). \end{array}$$

Thus, we get

$$k^{*}\left(t\right)=\frac{I^{*}\left(t\right)\left(\lambda_{2}\left(t\right)-\lambda_{4}\left(t\right)\right)}{\omega_{k}}.$$

Since $\left(h^{*},\ell^{*},k^{*}\right)\in U_{ad}$, the optimal control functions *h*∗, *ℓ*∗ and *k*∗ are expressed as in formulas (15), (16) and (17), respectively. The adjoint system is derived from (14) at *y* = *y*∗ and (*h*, *l*, *k*) = (*h*∗, *ℓ*∗, *k*∗) as follows:

$$\begin{array}{ll} \lambda_{1}^{\prime }\left(t\right) & ={\left(-\frac{\partial H\left(t\right)}{\partial S\left(t\right)}\right|}_{y=y^{*},\;h=h^{*},\;\ell =\ell^{*},\;k=k^{*}}\\ & =\left(\mu +h^{*}\left(t\right)+\beta I^{*}\left(t\right)+\bar{\beta }Q^{*}\left(t\right)\right)\lambda_{1}\left(t\right)-\left(\beta I^{*}\left(t\right)+\bar{\beta }Q^{*}\left(t\right)\right)\lambda_{2}\left(t\right)-h^{*}\left(t\right)\lambda_{3}\left(t\right),\\ \lambda_{2}^{\prime }\left(t\right) & ={\left(-\frac{\partial H\left(t\right)}{\partial I\left(t\right)}\right|}_{y=y^{*},\;h=h^{*},\;\ell =\ell^{*},\;k=k^{*}}\\ & =\left(\mu +\alpha +k^{*}\left(t\right)-\beta S^{*}\left(t\right)\right)\lambda_{2}\left(t\right)+\beta S^{*}\left(t\right)\lambda_{1}\left(t\right)-k^{*}\left(t\right)\lambda_{4}\left(t\right)-\omega_{i},\\ \lambda_{3}^{\prime }\left(t\right) & ={\left(-\frac{\partial H\left(t\right)}{\partial P\left(t\right)}\right|}_{y=y^{*},\;h=h^{*},\;\ell =\ell^{*},\;k=k^{*}}\\ & =\left(\mu +\ell^{*}\left(t\right)\right)\lambda_{3}\left(t\right)-\ell^{*}\left(t\right)\lambda_{1}\left(t\right),\\ \lambda_{4}^{\prime }\left(t\right) & ={\left(-\frac{\partial H\left(t\right)}{\partial Q\left(t\right)}\right|}_{y=y^{*},\;h=h^{*},\;\ell =\ell^{*},\;k=k^{*}}\\ & =\left(\mu +\bar{\alpha }\right)\lambda_{4}\left(t\right)+\bar{\beta }S^{*}\left(t\right)\lambda_{1}\left(t\right)-\bar{\beta }S^{*}\left(t\right)\lambda_{2}\left(t\right). \end{array}$$

This conclude the proof. □
